# Lipoprotein Association Fluorometry (LAF) as a Semi‐Quantitative Characterization Tool to Assess Extracellular Vesicle‐Lipoprotein Binding

**DOI:** 10.1002/jev2.70172

**Published:** 2025-09-30

**Authors:** Raluca Ghebosu, Jenifer Pendiuk Goncalves, Nur Indah Fitri, Dalila Iannotta, Mohammad Farouq Sharifpour, Elaina Coleborn, Alex Loukas, Fernando Souza‐Fonseca‐Guimaraes, Joy Wolfram

**Affiliations:** ^1^ Australian Institute for Bioengineering and Nanotechnology The University of Queensland Brisbane Queensland Australia; ^2^ School of Chemical Engineering The University of Queensland Brisbane Queensland Australia; ^3^ Australian Institute of Tropical Health and Medicine James Cook University Cairns Queensland Australia; ^4^ Frazer Institute, Faculty of Medicine The University of Queensland Brisbane Queensland Australia

**Keywords:** bad cholesterol, corona, exosomes, LDL, microvesicles, VLDL

## Abstract

Extracellular vesicles (EVs) are biological nanoparticles that play important roles in (patho)physiological processes and are promising new therapeutic and diagnostic tools. Recent evidence suggests that other circulating biological nanoparticles, primarily lipoproteins, bind to EVs, changing their biological identity. Such binding has been demonstrated with complex qualitative techniques, such as cryogenic transmission electron microscopy. There is a need to rapidly and simply quantify EV‐lipoprotein binding, as such complexes could have major implications for EV biology and medical applications. This study developed lipoprotein association fluorometry (LAF; based on fluorescent lipophilic indocarbocyanine dyes), as a first‐of‐its‐kind, simple and quick assay to assess EV binding to lipoproteins. The LAF assay was validated with synthetic nanoparticles, small molecules, polymers and proteins that display known interactions with lipoproteins. The LAF assay demonstrates that EVs from various human and non‐human (nematode and bacteria) sources bind to very‐low‐density lipoprotein (VLDL) and low‐density lipoprotein (LDL). Notably, EVs derived from cancerous cells displayed substantially increased binding to VLDL, LDL and plasma compared to EVs from normal cells. Additionally, the LAF assay revealed that EVs from metastatic cancer cells bound to VLDL to a greater extent than those from corresponding patient‐matched non‐metastatic cancer cells. On the contrary, EVs displayed minimal binding to high‐density lipoprotein (HDL). Taken together, the LAF assay is capable of measuring EV‐lipoprotein binding in a simple, rapid and semi‐quantitative manner, leading to new opportunities to probe EV biology and develop novel therapeutics, and diagnostics.

## 1 Introduction

Extracellular vesicles (EVs), are cell‐released nanoparticles with one or several lipid bilayers (Broad et al. 2023) and internal/external biomolecular cargo, such as nucleic acids, proteins and glycans (Hunter et al. 2008; Bastos‐Amador et al. 2012; Pendiuk Goncalves et al. 2023; Walker et al. 2020). EVs play a prominent role in (patho)physiological processes, mediating a wide range of functions in the body (Buzas 2023; Kalluri and McAndrews 2023; Ali et al. 2020). For example, cancer‐derived EVs can facilitate metastasis and immunoevasion (Marar et al. 2021; Xu et al. 2018; Chen et al. 2023; Pendiuk Goncalves et al. 2023; Coleborn et al. 2025). EVs are progressing through the translational pipeline as next‐generation therapeutic and diagnostic agents (Iannotta et al. 2021; Hu et al. 2021; Beetler et al. 2023; Ghodasara et al. 2023). However, interactions between EVs and other biological nanoparticles in biofluids remain poorly understood. Lipoproteins are biological nanoparticles that outnumber EVs by several orders of magnitude in the circulatory system (Simonsen 2017), indicating that EVs frequently encounter lipoproteins. Lipoproteins are comprised of a hydrophobic core consisting of triglycerides and cholesterol esters, and a phospholipid monolayer containing unesterified cholesterol, and apolipoproteins (Gotto et al. 1986). Lipoproteins are primarily involved in cholesterol transport between organs, but also carry other endogenous and exogenous molecules, including lipids and nucleic acids (Busatto et al. 2020; Ghebosu et al. 2024). The five main types of lipoproteins are chylomicrons, intermediate‐density lipoproteins (IDL), very low‐density lipoproteins (VLDL), low‐density lipoproteins (LDL) and high‐density lipoproteins (HDL), which differ in size, density and composition (Gotto et al. 1986). Many cells, including endothelial cells, express receptors that bind to lipoproteins, such as LDL receptor, VLDL receptor, scavenger receptor B1 (SR‐B1), activin receptor‐like kinase 1 (ALK1), LDL receptor related protein (LRP) 1,5 and 6, ATP‐Binding cassette transporter (ABC)A1, and ABCG1, many of which enable lipoproteins to enter and exit the circulation through transcytosis to deliver cargo to target cells (Busatto et al. 2020; Ghebosu et al. 2024; Amruta et al. 2023; Iannotta et al. 2024; Go and Mani 2012; May et al. 2007; Zhang et al. 2018).

Evidence is emerging that lipoprotein binding changes the biological identity of EVs, impacting extracellular/intracellular transport and effects on recipient cells (Ghebosu et al. 2024). Lipoprotein‐EV complexes can be transported together (Pham et al. 2023) and have been postulated to aid EVs in crossing the endothelium by using lipoprotein transcytosis pathways (Amruta et al. 2023; Iannotta et al. 2024). Studies have shown that mixing EVs with lipoproteins changes the levels of cellular internalization (Busatto et al. 2020) and substantially alters EV‐induced cytokine secretion in recipient cells (Busatto et al. 2022). EVs are also emerging as next‐generation drug delivery systems (Witwer and Wolfram 2021). making pharmacokinetics an important consideration. In small molecule drug discovery, lipoprotein binding is a key factor that is assessed for optimal pharmacokinetics (Chung and Wasan 2004; Wasan et al. 2008). Lipoprotein binding also affects targeting, biodistribution and stealth effects of synthetic nanoparticles (Butcher et al. 2016; Prawatborisut et al. 2022; Jiang et al. 2022; Gao and He 2014). Taken together, the aforementioned studies indicate the importance of assessing EV‐lipoprotein binding. However, in cases where EVs overlap in size with lipoproteins, such as VLDL, assessment of physical interactions becomes technically challenging, and methods to quantitatively and precisely measure binding between EVs and lipoproteins are lacking. Previous studies using complex and qualitative methods, such as cryogenic transmission electron microscopy (cryo‐TEM), demonstrated that EVs interact with lipoproteins in both homeostasis (Busatto et al. 2022; Lozano‐Andrés et al. 2023; Sódar et al. 2016) and disease states (cancer and viral infections) (Pham et al. 2023; Busatto et al. 2020). In particular, binding of plasma EVs to VLDL‐like particles has been reported. (Busatto et al. 2022) However, comprehensive assessment of lipoprotein binding to different EV subtypes has not previously been reported. Proteomics‐based studies have demonstrated binding of EVs to various apolipoproteins (Tóth et al. 2021), a key protein component of lipoproteins. However, such studies are unable to distinguish between EV binding to the protein components of lipoproteins versus intact lipoproteins. Therefore, there is a critical need for a simple, rapid and quantitative way to evaluate EV binding to intact lipoproteins, as this can facilitate understanding of EV biology and the development of EV‐based products.

In this study, we have developed lipoprotein association fluorometry (LAF) as a first‐of‐its‐kind, simple and time‐efficient semi‐quantitative assay to accurately predict EV binding to lipoproteins, including the identification of interactions that correlate with pathological states, such as cancer. EVs from human, plant, nematode and bacteria sources were assessed. The LAF assay was also validated with synthetic nanoparticles, polymers, proteins, peptides and small molecules with known interactions with lipoproteins, demonstrating broad applicability. Overall, the LAF assay overcomes labour intensive and time‐consuming qualitative methods that rely on specialized equipment, such as cryo‐TEM, providing a new semi‐quantitative characterization tool to rapidly assess lipoprotein interactions that are likely to change the biological identity of EVs.

## 2 Methods

### 2.1 Lipoprotein, Protein, Small Molecule and Polymer Preparation

Human VLDL (Sigma‐Aldrich/Merck, 437647) was diluted in phosphate buffer saline (PBS, pH 7.4, Gibco/Thermo Fisher Scientific, 10010‐031), stored at 4°C, and used within 1.5 months of opening. Human LDL (Sigma‐Aldrich/Merck, SAE0053) and HDL (Sigma‐Aldrich/Merck, L1567) were stored at −80°C for up to 2 months and 4°C for up to 2 weeks, respectively, prior to preparation for the assay. Lipoproteins were isolated from human plasma using proprietary methods and the manufacturer reported that the purity of VLDL and HDL were greater than 95% (assessed by electrophoresis), aligning with standards for analytical materials (Portoghese 1999, 2009). LDL purity was also confirmed by the manufacturer via electrophoresis. HDL and LDL have a quality rating of MQ200 and VLDL has a rating of MQ100. VLDL obtained from a different vendor was also used to validate the LAF assay (Abcam, AB91126). The fluorescent lipophilic indocarbocyanine dye, 1,1′‐dioctadecyl‐3,3,3′,3′‐tetramethylindocarbocyanine perchlorate (DiI), was acquired as part of the Vybrant multicolor cell‐labelling kit (Thermo Fisher Scientific, V22889) and diluted in ultra‐pure water (Invitrogen, 10977015) prior to use. Polyethylene glycol (PEG) 6000 (Sigma‐Aldrich/Merck, US1528877) was dissolved in PBS at a 50% w/v concentration. Lipoproteins were used directly or first diluted in PBS. Warfarin sodium salt (Sigma‐Aldrich/Merck, PHR1435‐1G) and tissue plasminogen activator (Sigma‐Aldrich/Merck, T0831) were diluted in ultra‐pure water (Invitrogen, 10977015). Cytochalasin D (Sigma‐Aldrich/Merck, C8273) was prepared and diluted in CryoMACS dimethyl sulfoxide (DMSO, Miltenyi Biotec, 170‐076‐303). The polymer, pre‐hydrolyzed styrene‐maleic acid copolymer 2:1 (SMA, Sigma‐Aldrich/Merck, SAE0062), was dissolved in 20 mM of 7.4, 4‐(2‐hydroxyethyl)‐1‐piperazineethanesulfonic acid (HEPES; Sigma‐Aldrich/Merck, H4034) with 100 mM sodium chloride (Sigma‐Aldrich/Merck, S9888) at pH 7.4 at a concentration of 2.2 mg/mL. Lipopolysaccharides (LPS, Sigma‐Aldrich/Merck, L2630) were dissolved in PBS to a final concentration of 1 mg/mL and stored at −80°C.

### 2.2 Synthetic Nanoparticle Preparation

Pegylated lipid nanoparticles were acquired from The University of Queensland BASE mRNA facility (service provider). The Pegylated lipid nanoparticles were manufactured by microfluidic mixing with the NanoAssemblr Ignite+ by Precision NanoSystems. Pegylated lipid nanoparticles were comprised of SM‐102 (Sapphire Biosciences, 33474), cholesterol (Sapphire Biosciences, 700100P‐100MG‐A‐036), 1,2‐dimyristoyl‐rac‐glycero‐3‐methoxypolyethylene glycol‐2000 (DMG‐PEG 2000, Sapphire Biosciences, 33945) and dimyristoyl glycerol, and 1,2‐distearoyl‐sn‐glycero‐3‐phosphocholine (DSPC, Sigma‐Aldrich/Merck, P1138) at a molar ratio of 50:38.5:1.5:1. Liposomes were prepared using the thin‐film hydration method and extrusion, as previously described (Wolfram et al. 2016) with modifications. Briefly, 10 mg of 1,2‐dimyristoyl‐*sn*‐glycero‐3‐phosphorylcholine (DMPC) (Sigma‐Aldrich/Merck, 850345P) was dissolved in 1 mL of a 3:1 v/v chloroform and methanol mixture into a round bottom flask. The resulting film was obtained by removing the organic solvent with Rotavapor (V‐100 Buchi, Switzerland) at 40°C while gradually decreasing the pressure to 40 mBar. To remove any trace of organic solvents, the film was stored overnight at room temperature in a fume hood. 1 mL of pH 7.4, 0.22 µM filtered HEPES (Sigma‐Aldrich/Merck, H4034) buffer was used to reconstitute the lipid films, which were then extruded sequentially through polycarbonate membranes with 800, 600, 400, 200 and 100 nm pores (Genizer, GE25562218; GE25561420; GE25561418; GE25561118; GE25560620) with a drain disc as support (Genizer, GE 21514125) using a jacketed liposome extruder (Genizer).

### 2.3 Human Cell Culture and Production of Conditioned Cell Culture Media

MDA‐MB‐231 TGL (Minn et al. 2005), MDA‐MB‐231‐BrM‐831 (Minn et al. 2009), MDA‐MB‐231‐Lm2‐4175 (Minn et al. 2005) and MDA‐MB‐231‐BoM‐1833 (Kang et al. 2003) metastatic human breast cancer cells were acquired from Memorial Sloan Kettering Cancer Center in the United States. All MDA cell lines, MCF‐7 poorly metastatic human breast cancer cells (ATCC, HBT‐22), and HEK293T human embryonic kidney cells (ATCC, CRL‐3216) were cultured in high glucose Dulbecco's modified Eagle's medium (DMEM, Sigma‐Aldrich/Merck, D5796). HOS non‐metastatic human osteosarcoma cells (ATCC, CRL‐1543) and 143B metastatic human osteosarcoma cells (ATCC, CRL‐8303) were cultured in Eagle's minimum essential medium (EMEM, Sigma‐Aldrich/Merck, M5650). NK‐92 human cancerous natural killer cells (ATCC, CRL‐2407) were cultured in Roswell Park Memorial Institute 1640 (RPMI, Gibco, 11875) medium. For cell maintenance, all media were supplemented with 10% fetal bovine serum (FBS, Gibco, 26140‐079) and 100 U/mL of penicillin and 100 µg/mL of streptomycin (Gibco, 15140‐122). Osteosarcoma cells (HOS and 143B) were further supplemented with 1 mM sodium pyruvate (Sigma‐Aldrich/Merck, S8636) and 2 mM L‐glutamine (Sigma‐Aldrich/Merck, G6784). NK‐92 cells were further supplemented with 300 IU/mL recombinant interleukin‐2 (IL‐2, PeproTech, 200‐02). ASC52telo, human hTERT immortalized adipose‐derived mesenchymal stromal cells (MSC, ATCC, SCRC‐4000) were cultured in mesenchymal stem cell basal medium for adipose, umbilical and bone marrow‐derived mesenchymal stem/stromal cells (ATCC, PCS‐500‐030) and supplemented with mesenchymal stem cell growth kit for adipose and umbilical‐derived mesenchymal stem/stromal cells—low serum (ATCC, PCS‐500‐040). All cells were incubated at 37°C in 5% CO_2,_ and cell viability was determined by trypan blue exclusion (Gibco, 15250061). The use of commercial human cell lines is approved under The University of Queensland's Human Research Ethics Approval number 2022/HE000725. Primary human natural killer cells were isolated from peripheral blood mononuclear cells, supplied by Australian Red Cross Lifeblood and approved by The University of Queensland's Human Research Ethics Approval number 2023/HE000027. Peripheral blood mononuclear cells were first isolated using Ficoll density gradient (Cytivia, 17144003) and LeucoSep tubes (Interpath, 227290_PK). Natural killer cells were then acquired from peripheral blood mononuclear cells by negative selection using MojoSort Human NK Cell Isolation Kit (BioLegend, 480053), as per manufacturer's instructions. Primary natural killer cells were cultured in NK MACS Medium (Miltenyi Biotec, 130‐114‐429), supplemented with 5% AB human serum (Sigma‐Aldrich/Merck, H2667), 100 U/mL of penicillin and 100 µg/mL of streptomycin (Gibco, 15140‐122), 5 ng/mL interleukin‐15 (IL‐15, Peprotech, 200‐15) and 500 IU/mL recombinant IL‐2 (PeproTech, 200‐02). All commercial cell lines were confirmed to be negative for mycoplasma.

Prior to EV isolation, cells were grown to 90% confluency in 175 cm^2^ flasks and washed twice with PBS. Most cells were serum starved for 24 h prior to isolation to minimize contamination from serum‐derived EVs and lipoproteins. HEK293T and NK‐92 cells were cultured for 24 and 18 h, respectively, in exosome‐depleted FBS (Gibco, A2720801) instead of starvation. Exosome‐depleted FBS is depleted of both EVs and lipoproteins (Busatto et al. 2022). Primary natural killer cells were grown in 10% EV/lipoprotein‐depleted plasma for 24 h prior to tangential flow filtration. EV/lipoprotein depletion of crude human plasma was performed by incubating plasma with a 15% w/v PEG 6000 (Sigma‐Aldrich/Merck, US1528877). Plasma samples were then vortexed and incubated for 1 h at 4°C before centrifugation at 500 × *g* for 10 min. The supernatant was then collected and filtered through a 0.22 µm filter (Sigma‐Aldrich/Merck, SLGS033SS). Aliquots of EV/lipoprotein‐depleted plasma were stored at −80°C. Human plasma was obtained from the Australian Red Cross Lifeblood, under the University of Queensland's Human Research Ethics Approval number 2022/HE000652. For EV isolation, 130–900 mL of the conditioned medium was collected from cells and centrifuged at 800 × *g* and 4°C for 30 min. The supernatant was immediately used for EV isolation using tangential flow filtration.

### 2.4 Tangential Flow Filtration and Diafiltration for Isolation of Human EVs

EVs were isolated from conditioned cell culture media by tangential flow filtration and diafiltration using a KrosFlo KR2i TFF System (Repligen) under sterile conditions (Busatto et al. 2020; Tian et al. 2020; Busatto et al. 2021; Wang et al. 2021; Busatto et al. 2018). Samples were processed using sterile hollow fibre modified polyethersulfone membranes with a 0.65 µm (Repligen, D02‐E65U‐07‐S) and 750 kDa (Repligen, D02‐E750‐05‐S) cut‐off to remove large cell debris and non‐EV associated biomolecules, respectively. Filters were washed with 3 mL/cm^2^ of filter area of PBS prior to processing and washed with the same volume of 0.1 M sodium hydroxide (NaOH, Sigma‐Aldrich/Merck, 795429) followed by PBS after use. The input flow rate was 130 mL/min for the first filter and between 50 and 65 mL/min for the second filter, ensuring the shear rate remained below 2000/s to protect against damage and maintain the structural integrity of EVs (Busatto et al. 2018). Samples were diafiltered six times in a sterile cryoprotective sucrose buffer (5% sucrose, 50 mM Tris and 2 mM MgCl_2_ (Walker et al. 2022)) and concentrated using the aforementioned flow and shear rate parameters. Sucrose buffer was sterilized by filtration through a 0.22 µm vacuum filter (Corning, 431118) and purified using TFF with a 50 kDa (Repligen, D02‐E050‐05‐S) filter prior to use. Samples were aliquoted and stored at −80°C.

### 2.5 Spirulina Cultures

Xenic spirulina microalgae stock (*Limnospira maxima*) was obtained from a commercial source (Spirulina Grow Co., Australia). The stock was maintained and expanded in Zarrouk's medium (Morist et al. 2001) within a custom‐built photobioreactor at room temperature, under constant illumination of 150 µE blue‐shifted white light from adjustable light‐emitting diode (LED) lights (Aqua Illumination Hydra 64 HD) with air injection for agitation.

To isolate pure spirulina EVs as previously described (Sharifpour et al. 2024), an axenic culture was produced and grown in Zarrouk's medium supplemented with 100 µg/mL kanamycin (Sigma‐Aldrich, 60615) until an optical density of 0.1 at 680 nm was reached. The optical density of spirulina cultures was measured in 48 well plates, with each containing 900 µL volume, using FLUOstar Omega (BMG LabTech). Spirulina filaments were pelleted by centrifugation at 1000 × *g* for 10 min at room temperature and resuspended in 10 mL of sterile MilliQ water. The concentrated Spirulina suspension was sonicated for 5 min and 100–200 µL aliquots were spread onto Zarrouk's agar medium in Petri dishes (Zarrouk's medium with 1.5% agar, Sigma Aldrich, 05040) and sealed with parafilm to prevent desiccation. The petri dishes were incubated for 4 weeks at room temperature under continuous light. Single colonies were then selected from the plates and transferred into new plates and incubated at room temperature under consistent light. When the optical density at 680 nm reached 0.3, approximately 3 mL of the axenic spirulina culture was transferred to 5 L of fresh medium and incubated under the previously described conditions.

For EV isolation, 2.25 L of the axenic spirulina culture at an optical density of 1.3 at 680 nm was subjected to sequential low‐velocity centrifugation steps (1000 × *g*, 2000 × *g*, 4000 × *g* and 10,000 × *g* for 11 min at 4°C) to collect the supernatant and remove large cellular debris. The supernatant was then vacuum filtered using a 0.45 µm polyethersulfone (PES) filter system (Corning, 430516) and the flow‐through, containing the EVs, was concentrated approximately 10‐fold using Vivaspin 20 filters with a 100 kDa cut‐off (Sigma‐Aldrich, GE28‐9323‐63), following the manufacturer's protocol. Subsequently, high‐velocity centrifugation of 200,000 × *g* for 70 min at 3°C was performed using an Optima MAX‐XP ultracentrifuge equipped with an MLA‐50 rotor and OptiSeal tubes (Beckman Coulter, 361625) and the pellet was resuspended in chilled PBS. To ensure the complete removal of soluble material, the ultracentrifugation step was repeated. The resulting crude EV pellet was resuspended in 2 mL of chilled PBS and EVs were further isolated by size‐exclusion chromatography (details below, section 2.8).

### 2.6 Hookworm Cultures

*Nippostrongylus brasiliensis* hookworms were grown and maintained in vivo, as previously described (Giacomin et al. 2008). Briefly, faecal cultures from 2‐week‐old rats were used to prepare infective larvae (L3). Three thousand L3 were subcutaneously injected into Sprague–Dawley rats (*Rattus norvegicus*) and adult worms were recovered from the small intestines 8 days post‐infection. The recovered adult worms were washed in PBS containing 5× antibiotic/antimycotic (AA, Gibco, Thermo Fisher, 15240096) and cultured for 7 days in RPMI medium supplemented with 1× AA and 1× GlutaMAX (Gibco, Thermo Fisher, 35050061) in 24‐well plates at a density of 500 worms per well at 37°C and 5% CO_2_. The media obtained during the first 4 h of parasite culturing was discarded. Excretory/secretory products were then collected daily, pooled and subjected to sequential differential centrifugation at 500 × *g*, 2000 × *g* and 4000 × *g* for 30 min each to remove eggs and parasite debris.

For EV isolation the media was concentrated using Vivaspin 20 filters with a 100 kDa cut‐off. The concentrated media then underwent high‐velocity ultracentrifugation at 170,000 × *g* for 90 min at 4°C using an Optima MAX‐XP ultracentrifuge with an MLA‐50 rotor and OptiSeal tubes. The resulting crude EV pellet was resuspended in chilled PBS and further isolated by size‐exclusion chromatography (please see details below, section 2.8).

### 2.7 Orange Sample Preparation

Oranges (2 kg; *Citrus sinensis*) were washed twice with tap water and juiced to yield approximately 800 mL of orange juice. The harvested juice underwent sequential low‐velocity centrifugation of 1000 × *g*, 2000 × *g* and 4000 × *g* for 10 min each at 4°C to collect the supernatant and remove large cellular debris. The supernatant was filtered through Whatman filter paper grade 1 (Sigma Aldrich, WHA1001090), followed by vacuum filtration through a 0.45 µm PES filter at 4°C. Approximately, 180 mL of the filtered juice was ultracentrifuged at 170,000 × *g* for 90 min at 4°C using an Optima MAX‐XP ultracentrifuge with an MLA‐50 rotor and OptiSeal tubes, resulting in a crude EV pellet which was resuspended in 1 mL of 20 µM Tris‐HCl solution by vigorous vortexing for more than 10 min (Stanly et al. 2016). The resuspended EVs were combined and ultracentrifuged again under the same conditions. The final resulting crude EV pellet was resuspended in chilled PBS and EVs were further isolated by size‐exclusion chromatography (details below, section 2.8).

### 2.8 Size‐Exclusion Chromatography for Isolation of Non‐Human EVs

EVs from spirulina, orange and hookworm were isolated by size‐exclusion chromatography. EV pellets were fractionated by size‐exclusion chromatography using a qEVoriginal isolation column (Gen 2, 35 nm) mounted on an automatic fraction collector (AFC; Izon), as per manufacturer's instructions. Fractions 2, 3 and 4 (out of 8) were collected as the purified EV sample.

### 2.9 Nanoparticle Tracking Analysis

Nanoparticle tracking analysis was performed using a NanoSight NS300 (Software NTA 3.4 Build 3.4.4; Malvern Panalytical Ltd, Malvern) equipped with a 405 nm laser. Samples were diluted in ultrapure water (Sigma‐Aldrich/Merck, W4502). EVs were diluted between 1:10 and 1:50 in water based on sample concentration to ensure particles per frame were between 40 and 100, as per manufacturer guidelines. Particle concentration and size distribution were analyzed from three 1‐min videos recorded using a detection threshold of five, camera level of 10, and a continuous syringe pump flow rate of 40 µL/min.

EVs used in the LDL, HDL and plasma studies were measured using a NanoSight Pro (Malvern Panalytical Ltd, Malvern), equipped with a 642 nm laser and diluted 1:50 in water. Automatic camera, focus and number of frame settings, and a flow rate of 1.5 µL/min was used. Due to differences in the detection sensitivity between the two pieces of equipment, a correction factor of 0.5 was applied to all EV concentrations measured by the NanoSight Pro.

### 2.10 Dynamic Light Scattering and Laser Doppler Micro‐Electrophoresis

Dynamic light scattering (particle size and concentration) and laser Doppler micro‐electrophoresis (zeta‐potential) were performed using a Zetasizer Ultra (Malvern, UK). Size distribution and concentration of liposomes and lipid nanoparticles were measured at 25°C by backscatter using 10 mm square polystyrene cuvettes (Sarstedt, 67.745). Refractive index of 1.45, medium viscosity of 0.8872 mPa.s, medium refractive index of 1.335 and medium dielectric constant of 78.5 were used. Zeta potential was measured at 25°C in disposable folded capillary cells (Malvern, DTS1070) using the in‐built Smoluchowsky model. Constant voltage was manually set at 50 mV and a maximum of 30 runs were performed per each analysis. Data were analyzed using Zetasizer Advance—ZS Xplorer v3.00 software (Malvern, UK). Samples were diluted 1:100–1:1000 (v/v) in isosmotic 5 mM NaCl, PBS solution at pH 7.4 to avoid multiple scattering phenomena.

### 2.11 Cryo‐TEM

Cryo‐TEM was used to identify phospholipid bilayers, a key authentication feature of EVs. EV samples were prepared for imaging using a Leica EM GP2 robotic vitrification system (Leica, Germany), under controlled temperature (22°C) and humidity (95%). Samples (3 µL) were dispersed onto a Lacey formvar carbon—Cu, 200 mesh grid (Electron Microscopy Services). Excess solution was mechanically blotted away for 2.5–3.5 s. Subsequently, samples were mechanically plunged into −182.8°C liquid ethane, to vitrify the sample. Samples were stored in liquid nitrogen prior to imaging using a Jeol Cryo ARM 300 (JEM‐Z300FSC) TEM equipped with a cold field emission gun and an Omega energy filter. Images were captured at a 300 kV acceleration voltage and 20 eV filter setting, with zero energy loss. Images were acquired under low‐dose conditions by a Gatan K3 direct detector camera and processed using the SerialEM software (Mastronarde 2005). Semiquantitative data of interactions between EVs and VLDL were determined by counting the number of EVs that had VLDL bound to the surface or were fused with VLDL. Cases with multiple VLDL particles attached to a single EV or multiple EVs to a single VLDL particle were counted as a single instance. Additionally, cases were excluded if the EV was only partially in frame and showed no binding within the observable region. Instances are displayed as percentages, that is, the sum of binding and fusion or fusion alone divided by the total number of EVs counted in 56 (HEK293T), 81 (MCF‐7), 88 (MDA‐MB‐231‐BoM‐1833), 49 (HOS) and 64 (143B) micrographs with a surface area of approximately 103.8 µm^2^ each. EVs can be distinguished from VLDL by the presence of a phospholipid bilayer and aqueous core, which is much lighter in colour than VLDL. VLDL particles display a single phospholipid monolayer, polygonal‐like faceted sides (Busatto et al. 2022), and a more electron‐dense (darker) centre.

### 2.12 Western Blot

Sample protein concentration was determined by a Peirce micro bicinchoninic acid (BCA) protein assay kit (Thermofisher, 23235) to allow for standardization of protein concentration. Samples were diluted in sucrose buffer and prepared in 1× Laemmli sample buffer (BioRad, 1610747) prior to heating for 5 min at 90°C. Samples (1.22 µg/well) or Precision Plus Protein Kaleidoscope Prestained Protein Standard (BioRad, 1610375) were loaded into NuPAGE 4%–12%, Bis‐Tris, 1.5 mm protein gels (Invitrogen, NP0321) and submerged in 1× NuPAGE running buffer (Invitrogen, NP0001) with NuPAGE antioxidants (Thermofisher, NP0005). Electrophoretic separation was performed at 120 V for 2–2.5 h using a XCell SureLock mini‐cell (Invitrogen, EI0001) and PowerPac basic power supply (BioRad, USA). Gels were removed and transferred using an XCell II blot module (Invitrogen, EI9051) with 1× NuPAGE transfer buffer (Invitrogen, NP00061) and NuPAGE antioxidants (Thermofisher, NP0005) at 200 mA for 1.5 h. A total of 10% methanol (Sigma‐Aldrich/Merck, 106018) was added for one gel and 20% for two gels.

Protein bands and successful transfer were assessed by Ponceau staining (Thermo Fisher Scientific, A40000279). Ponceau was washed using 1× Tris‐buffered saline and 0.1% Tween (TBST) (Thermofisher, 28360). Membranes were then blocked for 1 h at room temperature with 5% skim milk (w/v) on an orbital shaker. Membranes were incubated on a shaker overnight at 4°C with the following primary antibodies and dilutions in 1% w/v milk TBST: Alix (1:200, Cell Signaling, 2171S), calnexin (1:250, GeneTex, GTX112886), CD63 (1:250, Abcam, ab134045), CD81 (1:500, Santa Cruz, sc‐166029) or CD9 (1:250, Cell Signaling, 13174S). Following incubation, membranes were washed four times for 5 min with 1× TBST. Horseradish peroxidase (HRP)‐conjugated secondary antibodies anti‐rabbit IgG (New England Biolabs, 7074P2) and anti‐mouse immunoglobulin G (IgG, Thermo Fisher Scientific, 31430) were added at a 1:3000 dilution in 1% w/v milk TBST and incubated for 1 h at room temperature. Secondary antibodies were removed by washing three times for 5 min with 1× TBST. Membranes were incubated with SuperSignal West Dura Extended Duration Substrate (Thermo Fisher Scientific, 34075) and imaged using a ChemiDoc MP Imager (Bio‐Rad).

### 2.13 Labelling of VLDL With an Apolipoprotein B (apoB) Antibody

VLDL stock solution of 2.68 mg/mL (protein concentration) was diluted 1:200 in 2 mL and incubated for 1 h at 37°C. VLDL aggregates were then incubated with 1 µg/mL of apoB antibody (1:500, Proteintech, 20578‐1‐AP) overnight at 4°C. Excess apoB was removed by a 4h dialysis with stirring at room temperature and a 300 kDa dialysis membrane (Repligen, 131450), secured on both ends with dialysis clamps. The surrounding dialysis solution consisted of a 150 mM NaCl (Sigma‐Aldrich/Merck, S9888) and 0.01% EDTA (Thermo Fisher Scientific, R1021) that was sterile filtered through a 0.22 µm vacuum filter (Corning, 431118). This solution was selected due to it being the original storage solution of VLDL. The dialyzed solution was then further incubated overnight at 4°C with a donkey anti‐Rabbit Alexa Fluor 488 secondary antibody (1:500, Thermo Fisher Scientific, A‐21206). The sample was then dialyzed again in fresh NaCl/EDTA buffer to remove unbound secondary antibody. A total of 50 µL of the labelled VLDL solution was then incubated with 5 µM of the fluorescent lipophilic indocarbocyanine dye, DiD (Thermo Fisher Scientific, V22887, excitation 648 nm and emission 670 nm), for 1 h at 37°C to stain for hydrophobic lipid cores. The fully labelled mixture (3 µL) was loaded onto a glass slide (Westlab, 663‐248) and covered with a clear glass coverslip (Westlab, 663‐251). The fluorescence was then visualized using an ECLIPSE Ti2‐U Microscope (Nikon, Japan) equipped with a FITC (apoB) and Cy5 filter (DiD) and exposure setting of 1 s for 10× magnification and 300 ms for 20× magnification. A blank PBS solution without VLDL was labelled and imaged in parallel to confirm the successful removal of primary and secondary antibodies.

### 2.14 Preparation of LDL and HDL Samples

LDL and HDL samples were dialyzed using a 3.5 kDa dialysis tube (Sigma‐Aldrich/Merck, PURD35050) for 4 h at room temperature with stirring. To maintain sterile conditions during dialysis, a 150 mM NaCl (Sigma‐Aldrich/Merck, S9888) and 0.01% EDTA (Thermo Fisher Scientific, R1021) solution was sterile filtered through a 0.22 µm vacuum sterile filter (Corning, 431118) and used as the surrounding dialysis solution. The protein concentration of the dialyzed samples was measured by Peirce micro‐BCA protein assay kit (Thermofisher, 23235) and samples were stored at 4°C for up to 2 weeks.

### 2.15 Lipoprotein Association Fluorometry Assay

The semi‐quantitative LAF assay was performed in a 96‐well microplate format with a final volume of 50 µL in each well. Transparent 96‐well plates (Corning, 3598) were used for light microscopy images, and Nunc MicroWell 96‐Well Optical‐Bottom plates (Thermo Fisher Scientific, 165305) were used for fluorescence readings. All solutions were warmed to room temperature prior to use in the LAF assay. Reagents were added to each well in the following order: PBS, lipoproteins, test sample and DiI. For standard curve generation, various dilutions of the stock protein concentration of 1.83 mg/mL VLDL solution were prepared (1:10, 1:20, 1:50, 1:100, 1:200, 1:500, 1:1000 and 1:2000) in PBS in the wells. Various dilutions were also prepared for the 7.54 mg/mL (protein concentration) LDL and 3.69 mg/mL (protein concentration) HDL samples. The concentration of NaCl and EDTA buffer was not maintained for VLDL samples but was maintained for HDL and LDL samples. The VLDL standard curve values were confirmed to be unaffected by alterations in the salt concentration (data not shown). For fluorescence measurements, 5 µM of DiI was added to each well. Plates were then incubated for 1 h at either 37°C or room temperature.

Fluorescence was measured using a Tecan Infinite 200 plate reader (Tecan, Switzerland). A fluorescence intensity scan with an excitation wavelength of 528 nm was performed at 37°C to identify the ideal emission wavelength. Excitation and emission wavelengths of 528 and 576 nm, respectively, were identified as ideal with nine reads per well (3×3 format). Optimal fluorescent gain settings were used at 37°C, and this same gain setting was then used to assess the corresponding room temperature plate. Any non‐normalized pooled data that was performed on separate instances and/or being directly compared, maintained the same gain and wavelength settings. The fluorescence intensity of a well containing only DiI in the absence of lipoproteins was subtracted from each experimental group as background fluorescence.

To minimize reagent use, while still identifying differences within a broad dynamic range in response to test agents with known interactions (e.g. warfarin), protein concentrations of 37 µg/mL of VLDL and 151 µg/mL of LDL were used at a temperature of 37°C. In the case of HDL, a protein concentration of 37 µg/mL at room temperature was determined as optimal. Reagents were added to each well in the following order: PBS, lipoproteins, test sample, DiI. For warfarin, tissue plasminogen activator, cytochalasin D and LPS studies, drugs were diluted to 1 mg/mL concentrations and 5 µL of each drug was added to the assay (5 µg/well). A total of 5 µL of the respective diluent (water, DMSO or PBS) was added to DiI and lipoproteins as control wells. The same setup was performed for the warfarin standard curve studies, where 2.5 or 5 µL of various warfarin concentrations were added. All wells were adjusted to the same final volume (50 µL) and all data normalized to the appropriate controls, with corresponding volumes of diluent (water, DMSO or PBS) added.

For EVs or nanoparticle studies, all samples were diluted to a concentration of 1 × 10^10^ particles/mL in sucrose buffer and 5 µL was added to each well (1 × 10^7^ particles/well). Where EV concentration was below 1× 10^10^ particles/mL, volumes were adjusted to the matched particle number (5 × 10^7^ particles). *N. brasiliensis* EVs were diluted in PBS and had a concentration below 1 × 10^10^, therefore, the matched particle number (5 × 10^7^ particles), and 5 µL of sucrose buffer was added to ensure equal volumes of all solutes. The PEG groups contained various dilutions of a 50% w/v PEG solution in PBS. A total of 5 µL of sucrose buffer was also added to a lipoprotein control, free DiI and all PEG groups. Five µL of styrene maleic acid at a final concentration of 440 µg/mL was used (22 µg/well). In all cases, DiI was added at a final concentration of 5 µM to each well (2.5 µL of 100 mM stock per well), and PBS added to a final volume of 50 µL. Plates were incubated for 1 h at 37°C or room temperature and the fluorescence intensity was immediately measured as previously described. Donor, supplier and batch variations may result in lipoproteins having varying susceptibility to partitioning changes. In cases where lipoproteins are highly susceptible to aggregation, performing the assay at room temperature may yield improved results. A positive control that is known to display substantial binding to lipoproteins, that is, warfarin (Rosengren et al. 2012; Yacobi et al. 1976; Tokui et al. 1995), should be used to assess ideal incubation conditions.

Human plasma was obtained from healthy donors, and depleted of fibrin to prevent potential clotting. Fibrin depletion was achieved by exposing plasma to 5 IU/mL of bovine thrombin (Sigma‐Aldrich/Merck, T4648), diluted in 0.1% BSA (Sigma‐Aldrich/Merck, A8412). Samples were incubated for 10 min at room temperature with thrombin to allow for clot formation, then centrifuged at 10,000 × *g* for 10 min. The resulting supernatant (fibrin‐depleted plasma) was then utilized in the LAF assay. A standard curve was generated using various plasma concentrations (v/v) diluted in PBS. A concentration of 20% v/v plasma, incubated at 37°C for 1 h was evaluated with various nanoparticles and warfarin as previously detailed.

### 2.16 Statistical Analysis

Statistical analyses were performed in GraphPad Prism 9.4.1 software (Dotmatics). Statistical analyses were determined using one‐way analysis of variance (ANOVA) with Tukey's multiple comparison test. Statistical analysis for donor plasma samples was determined using two‐way ANOVA with Tukey's multiple comparison test (between donors) or Dunnett's multiple comparison test (compared to 100% control). Significance is displayed in the respective figure legends.

## 3 Results

### 3.1 Concentration‐ and Temperature‐Dependent Changes in VLDL Can Be Fluorometrically Quantified

**FIGURE 1:**
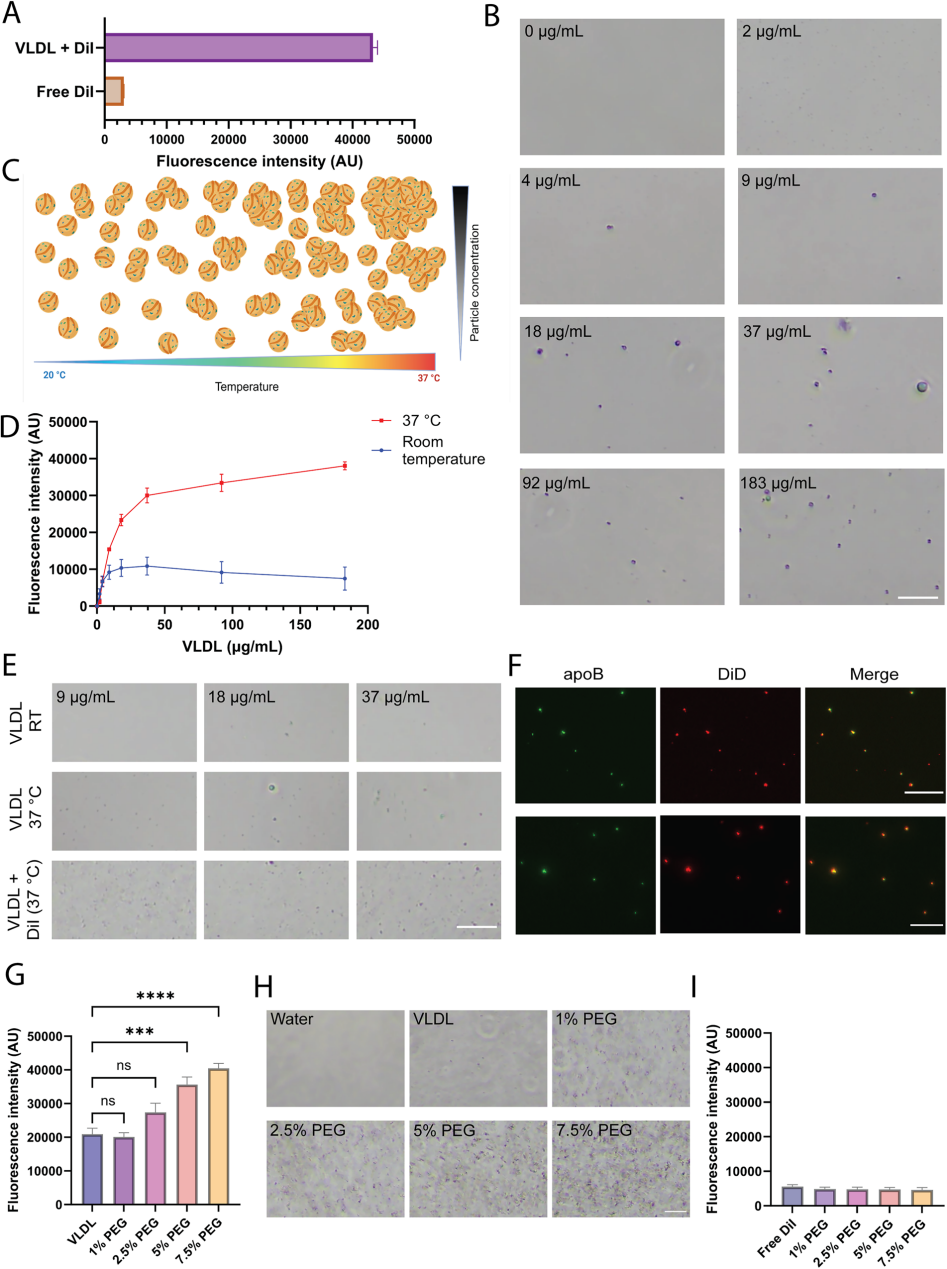
Temperature and concentration‐dependent aggregation of very low‐density lipoprotein (VLDL) can be quantified fluorescently. (A) Fluorescence intensity of the fluorescent lipophilic indocarbocyanine dye, DiI (1,1′‐dioctadecyl‐3,3,3′,3′‐tetramethylindocarbocyanine perchlorate), in the presence and absence of VLDL. (B) Light microscopy images of aggregates present at various VLDL protein concentrations incubated at 37°C for 1 h (in the absence of DiI). Scale bar, 50 µm. (C) Schematic representation of temperature and concentration‐dependent aggregation of VLDL. (D) Fluorescence intensity of various protein concentrations of DiI‐labelled VLDL incubated at 37°C and room temperature. (E) Light microscopy images of aggregates present at various VLDL protein concentrations incubated at room temperature or 37°C for 1 h in the presence/absence of DiI. Scale bar, 50 µm. (F) Fluorescence microscopy images of VLDL labelled with apolipoprotein B (apoB; green) and the fluorescent lipophilic indocarbocyanine dye, DiD (red). Scale bar, 100 µm (upper, 10× magnification) and 50 µm (lower, 20× magnification). (G) Fluorescence intensity of 37 µg/mL of VLDL (protein concentration) incubated with 1%, 2.5%, 5% and 7.5% polyethylene glycol (PEG). (H) Light microscopy images of VLDL aggregates present at increasing concentrations of PEG (in the presence of DiI). Scale bar, 50 µm. (I) Fluorescence intensity of various PEG concentrations incubated with DiI in the absence of VLDL. (B, G, I) The fluorescence intensity of free DiI was subtracted as a background signal. Bar graphs show mean of three representative measurements (D) or four (G, VLDL alone group) or six representative individual samples (G, I) + standard error of mean (SEM). Results were validated in at least three separate experiments. Differences in the fluorescence intensity between figures are due to differences in machine settings that are optimized for each experiment. Gain settings are maintained where appropriate to allow for direct comparisons between free DiI and VLDL with DiI. Statistical analysis performed by ordinary one‐way analysis of variance (ANOVA) with Tukey's multiple comparison test (G). ***p < 0.001; ****p < 0.0001. ns, not significant.

Recent evidence suggests that lipoprotein binding affects the biological identity of EVs including cell signalling and transport (Ghebosu et al. 2024). Among lipoproteins, VLDL displays prominent binding to EVs in human blood (Busatto et al. 2022). However, VLDL overlaps in size with EVs, making it challenging to assess binding due to an inability to remove unbound VLDL through size‐based methods. The goal of this study was to develop a simple assay to detect the formation of EV‐lipoprotein complexes without relying on the separation of components. This goal was accomplished by using fluorescent lipophilic indocarbocyanine dyes, such as DiI, which are known to intercalate in phospholipid layers. Such dyes display higher fluorescence intensity in hydrophobic/lipophilic compartments, such as lipoproteins (Figure 1A). We observed that higher concentrations of VLDL at 37°C induce aggregation (Figure 1B,C) and increase the fluorescence intensity of DiI (Figure 1D). This increase in fluorescence intensity is most likely due to changes in dye partitioning, which has previously been observed in lipid bilayer structures (Baumgart et al. 2007). In addition to changes in dye partitioning (which may also relieve self‐quenching), increased labelling efficiency at 37°C may be due to other temperature‐dependent effects, such as faster insertion kinetics and enhanced dye diffusion. It is worth noting that the presence of DiI further accelerated the aggregation of VLDL (Figure 1E). To confirm that these fluorescent aggregates contained VLDL, as opposed to dye‐only aggregates, staining for apoB (key protein component of VLDL) was performed. The results revealed co‐localization between the dye and apoB protein (Figure 1F). Taken together, the results indicate that the fluorescence‐based assay is able to measure temperature and concentration‐dependent changes in VLDL.

To further validate that changes in fluorescence intensity correlate with VLDL aggregation, the synthetic polymer PEG, which is known to precipitate/aggregate lipoproteins (crowding agent) (Viikari 1976), was added at various concentrations (1%–7.5%). Addition of PEG substantially induced lipoprotein aggregation and increased the fluorescence intensity of DiI (Figure 1G,H). Increased fluorescence likely reflects changes in DiI partitioning, with aggregation being correlative rather than causative. The fluorescence intensity remained unchanged with increasing concentrations of PEG in DiI control groups without VLDL (Figure 1I), suggesting that the increased fluorescence intensity resulted from changes in VLDL rather than interactions between the PEG and DiI. These findings collectively demonstrate the fluorometric ability of the assay to identify aggregation‐associated changes in VLDL.

### 3.2 LAF Assay Validation With Small Molecules and Proteins

**FIGURE 2:**
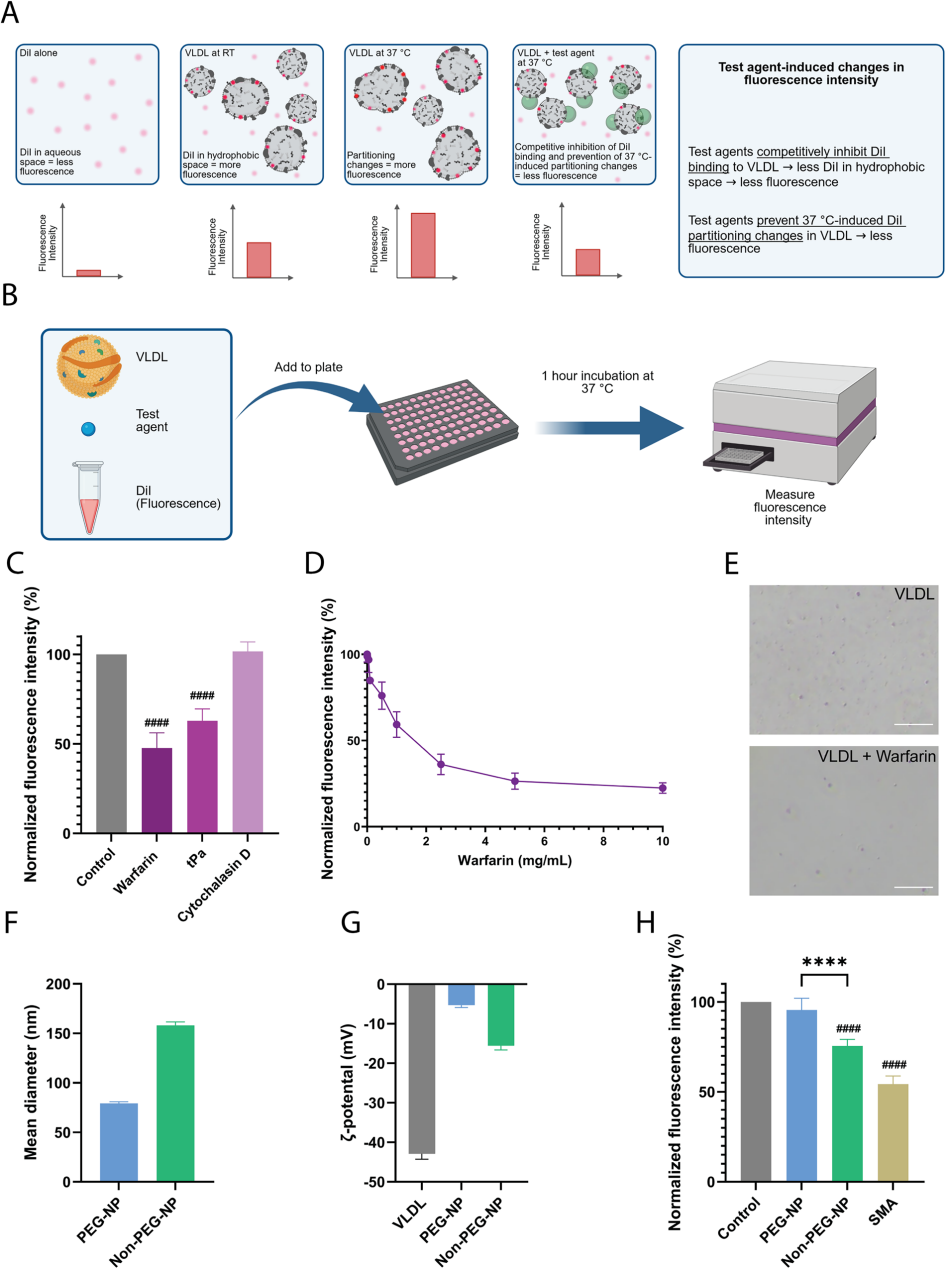
Lipoprotein association fluorometry (LAF) quantifies small molecule, protein, synthetic nanoparticle and polymer binding to VLDL. (A) Schematic illustration showing that binding of small molecules/proteins decreases the fluorescence intensity of DiI‐labelled VLDL. (B) Illustration of the LAF assay workflow. (C) Fluorescence intensity of VLD/DiI mixed with 5 µg of small molecules or proteins at 37°C. Warfarin (lipophilic small molecule) and tissue plasminogen activator (tPa; protein) are known to interact with lipoproteins, while cytochalasin D (small molecule) lacks known interactions with lipoproteins. (D) Concentration‐response curve to warfarin. (E) Light microscopy images of VLDL in the presence/absence of warfarin. Scale bar, 50 µm. Size (F) and zeta‐potential (G) of synthetic lipid‐based non‐pegylated nanoparticles (non‐PEG‐NP), pegylated nanoparticles (PEG‐NP) and VLDL assessed by dynamic light scattering (size) and laser Doppler microelectrophoresis (zeta potential). (H) Fluorescence intensity of VLDL/DiI with non‐PEG‐NP (known interactions with lipoproteins), PEG‐NP (PEG reduces interactions with lipoproteins) or a lipoprotein‐binding polymer (styrene maleic acid/SMA) (reduction indicates binding). Graphs show mean of three (F, G), four (D) or six (C, H) individual samples + SEM. The fluorescence intensity of the free DiI signal was subtracted from the data that were then normalized to the control group (VLDL/DiI alone, 100%). Results were validated in at least three separate experiments. Statistical analysis performed by ordinary one‐way ANOVA with Tukey's multiple comparison test. ****p < 0.0001 between groups and ^####^ *p* < 0.0001 compared to control.

We hypothesized that binding of molecules/macromolecules (test agents) to VLDL, prevents VLDL aggregation (and associated changes in DiI partitioning) and competes with DiI for VLDL binding, thereby, reducing the fluorescence intensity (Figure 2A). This reduction in fluorescence intensity could then be quantitatively measured, providing the foundation for the LAF assay, which requires minimal hands‐on time and a 1‐h incubation (Figure 2B). Using this method, clinically approved therapeutics that are known to bind to lipoproteins were assessed to determine whether VLDL fluorescence intensity was reduced. Specifically, the LAF assay was tested with warfarin, a lipophilic, small molecule that displays extensive binding to plasma proteins (Rosengren et al. 2012; Yacobi et al. 1976; Tokui et al. 1995) and tissue plasminogen activator, a protein that exhibits known physical interactions with VLDL and LDL (Dai et al. 2023; Romagnuolo et al. 2014; Simon et al. 1991). A non‐clinically approved small molecule, cytochalasin D, was also assessed, as there is no indication that this molecule binds to VLDL or other lipoproteins. A substantial decrease in fluorescence intensity was observed with the lipoprotein‐binding drugs, warfarin and tissue plasminogen activator (Figure 2C), while the fluorescence intensity remained unchanged with cytochalasin D (Figure 2C). To further validate that VLDL‐binding agents reduce fluorescence intensity, a concentration‐response assessment to warfarin was performed. The results indicate that higher concentrations of warfarin led to a more pronounced reduction in fluorescence intensity (Figure 2D). Light microscopy images demonstrate that the addition of warfarin at the concentration used in the LAF assay also reduces visible aggregation of VLDL (Figure 2E). To establish the minimum effective level of reagent use, every component of the assay (VLDL, DiI and warfarin) underwent serial dilutions. The results indicate that the warfarin‐induced reduction in fluorescence was halved at a fourfold dilution and was abolished at a 16‐fold dilution (2.3 µg/mL VLDL protein, data not shown). Taken together, in the case of small molecules and proteins, binding to VLDL could be fluorometrically measured, most likely due to test agents preventing temperature‐induced changes in VLDL partitioning (associated with aggregation and increased fluorescence intensity) or competitive inhibition of DiI binding.

### 3.3 LAF Assay Validation With Synthetic Nanoparticles and Polymers

To further validate the effectiveness of the LAF assay, we assessed its compatibility with synthetic nanoparticles and polymers that are known to interact with lipoproteins, Currently, complex methods, such as gradient ultracentrifugation, mass spectrometry and surface plasmon resonance, are required to assess nanoparticle interactions with lipoproteins, and this is generally only applicable for apolipoproteins (protein components of lipoproteins), not intact lipoproteins (Sebastiani et al. 2022; Klein 2007; Aggarwal et al. 2009). Therefore, there is a need to develop methods that measure nanoparticle binding to intact lipoproteins. Here, DMPC liposomes (without PEG) with a mean size of 158 nm and a zeta potential of −16 mV were assessed (Figure 2F,G), as multiple studies have shown that phosphatidylcholine liposomes bind to lipoproteins (Ćwiklińska et al. 2014; Nakhaei et al. 2021; Bonté and Juliano 1986; Tall et al. 1986). Synthetic lipid nanoparticles (with PEG) with a size of 79 nm and zeta potential of −5 mV were also assessed (Figure 2F,G). Pegylation of nanoparticles is known to reduce interactions with circulating components due to a stealth effect (Butcher et al. 2016). This effect is distinct from the effects of high concentrations of free PEG, which acts as a crowding agent (Viikari 1976), increasing the aggregation of VLDL (Figure 1G,H). Given the stealth effect of lipid‐associated PEG (Butcher et al. 2016), it was expected that the pegylated nanoparticles would bind less to VLDL than the non‐pegylated ones. The LAF assay results revealed that addition of non‐pegylated nanoparticles led to a substantial reduction in fluorescence intensity, indicating binding to VLDL, while the pegylated lipid nanoparticles did not (Figure 2H). The results cannot be attributed to electrostatic interactions, as the pegylated nanoparticles (zeta potential: −5 mV) would be more likely to interact with VLDL (zeta potential: −43 mV) than the non‐pegylated ones (zeta potential: −16 mV). Taken together, the LAF assay was capable of differentiating VLDL binding between synthetic nanoparticles known to substantially bind to lipoproteins and those designed to reduce lipoprotein binding through pegylation. Additionally, the LAF assay's capability to measure VLDL binding to a polymer, styrene maleic acid, which is known to substantially interact with lipoproteins (Iannotta et al. 2024), was evaluated. Styrene maleic acid caused a pronounced reduction in fluorescence intensity, indicating substantial binding and altered DiI partitioning in VLDL (Figure 2H). Overall, the developed simple and rapid (1 h incubation) LAF assay measured VLDL binding to a broad range of molecules/particles with known lipoprotein interactions, including small molecules, proteins, synthetic lipid nanoparticles and polymers.

### 3.4 LAF Quantifies EV and VLDL Binding

A major advantage of the developed LAF assay is that output measurements are based on changes to VLDL rather than measuring the bound/unbound agent of interest, enabling rapid and simple assessment with broad applicability. Following LAF assay validation with various molecules, macromolecules and synthetic nanoparticles, EVs from different sources were assessed. Identifying EV‐lipoprotein interactions could have major implications for understanding EV biology, as previous studies have shown that EVs bind to intact lipoproteins in healthy (Busatto et al. 2022; Lozano‐Andrés et al. 2023) and disease (Pham et al. 2023; Busatto et al. 2020) states. However, techniques to assess binding are complex, time consuming and often fail to differentiate binding of lipoproteins versus protein components (apolipoproteins). Furthermore, side‐by‐side comparisons and quantification of lipoprotein binding to various EV types are lacking due to technical difficulties in obtaining such data.

**FIGURE 3:**
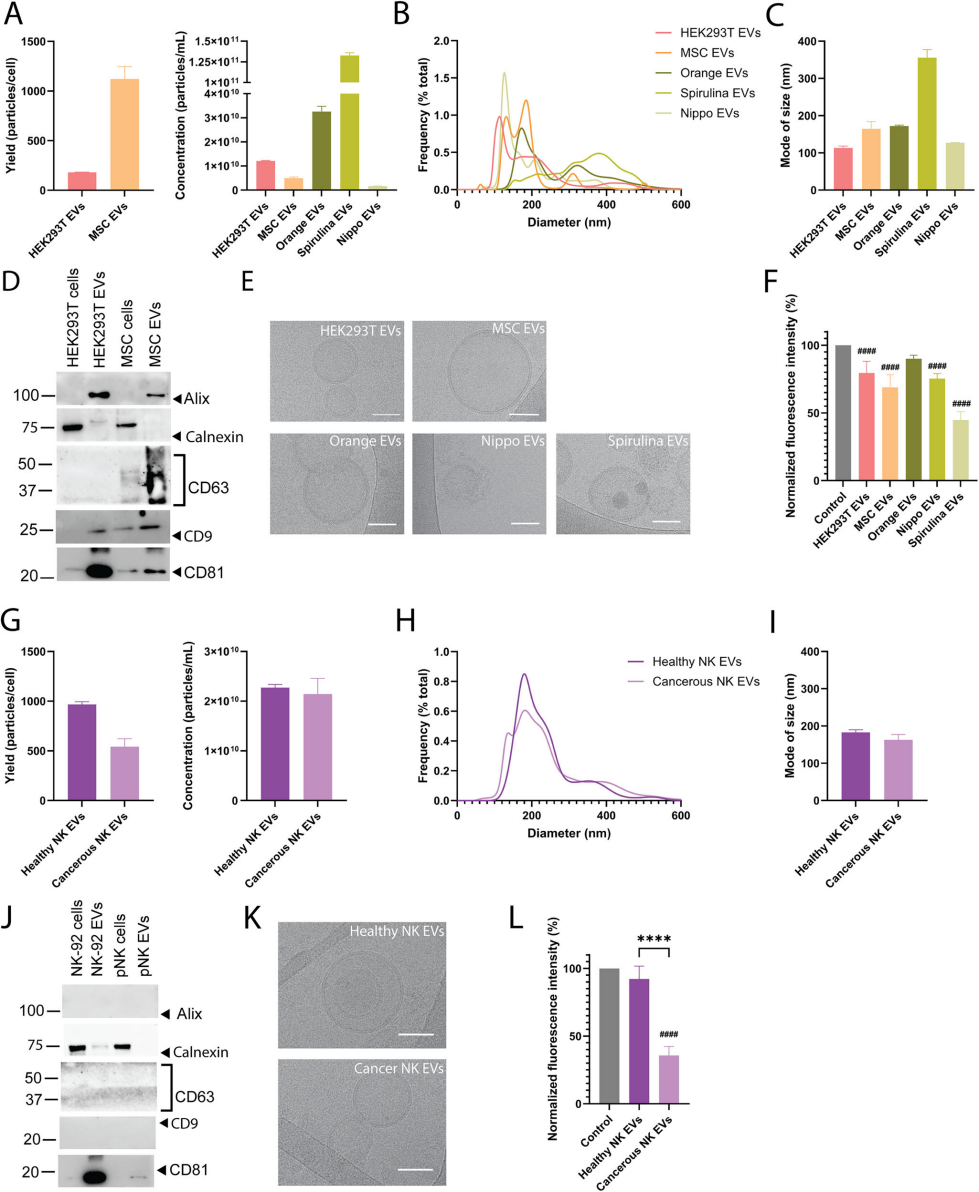
LAF quantifies VLDL binding to extracellular vesicles (EVs) from various origins and disease states. Concentration and yield (A), size distribution (B) and mode of size (C) of human embryonic kidney cell (HEK293T), human mesenchymal stromal cell (MSC), orange, *Nippostrongylus brasiliensis* (nematode, *Nippo*) and spirulina (cyanobacteria)‐derived EVs assessed by nanoparticle tracking analysis. (D) Western blot analysis of EV markers (alix, CD63, CD9, CD81) and contaminant marker (calnexin) in EVs and cell homogenates. (E) Cryogenic transmission electron microscopy (cryo‐TEM) images of isolated EVs. Scale bar, 50 nm. (F) Fluorescence intensity of VLDL/DiI with EVs (reduction indicates binding). Nanoparticle tracking analysis showing concentration and yield (G), size distribution (H) and mode of size (I) of heathy human primary natural killer (pNK) cell and human cancerous NK cell (NK‐92) EVs. (J) Western blot analysis of EV markers (alix, CD63, CD9, CD81) and contaminant marker (calnexin) in EVs and cell homogenates. (K) Cryo‐TEM images of isolated EVs. Scale bar, 50 nm. (L) Fluorescence intensity of VLDL/DiI with EVs (reduction indicates binding).Graphs show mean of three representative measurements of one sample + SEM (A–E, G–K) or six representative individual samples (F, L) + SEM. Results were validated in at least three separate experiments. The fluorescence intensity of free DiI was subtracted from the data that were then normalized to the control group (VLDL/DiI alone, 100%). Statistical analysis performed by ordinary one‐way ANOVA with Tukey's multiple comparison test. ****p < 0.0001 between groups and ^####^ *p* < 0.0001 compared to control.

The LAF assay was assessed with EVs isolated from various sources, including from human (HEK293T embryonic kidney cells and adipose‐derived mesenchymal stromal cells), plant (orange, *C. sinensis*), nematodes (*N. brasiliensis*) and bacteria (spirulina, *L. maxima*). The EVs were validated according to guidelines in the minimal information for studies of EVs (Théry et al. 2018; Welsh et al. 2024). Briefly, EV size and particle concentration were assessed by nanoparticle tracking analysis (Figure 3A–C). Western blot was performed on EVs to confirm the enrichment of EV markers (CD9, CD81 and C63) as well as the depletion of a contaminant marker (calnexin) (Figure 3D). Cryo‐TEM was performed to confirm the presence of phospholipid bilayers, a major authenticating factor of EVs (Figures 3E and S1A). All EVs except those from oranges bound to VLDL, with spirulina EVs displaying the highest extent of binding (Figure 3F). Spirulina has been reported to lower plasma cholesterol levels (Serban et al. 2016; Deng and Chow 2010), however, binding of spirulina EVs to lipoproteins has not previously been reported. Other studies have shown that EVs from human sources bind to lipoproteins (Pham et al. 2023; Busatto et al. 2020; Busatto et al. 2022; Lozano‐Andrés et al. 2023), but this is the first study to suggest that EVs from nematodes and bacteria also bind to lipoproteins. Human EVs were isolated by tangential flow filtration, while the spirulina, orange and nematode EVs were isolated by ultracentrifugation combined with size‐exclusion chromatography. EV isolation methods have been shown to alter the biomolecular identity of EVs (Wolf et al. 2022), suggesting that VLDL interactions may also vary based on the method. In this study, an isolation‐dependent pattern of VLDL binding was not apparent. Nevertheless, isolation methods should be considered when comparing different EV groups. Taken together, the LAF assay proved capable of measuring varying degrees of VLDL binding to various EV types in a rapid and simple fluorometric manner.

### 3.5 LAF Quantifies EV and VLDL Binding in Healthy Versus Cancerous States

Given the capability of measuring VLDL binding to EVs from a broad range of sources (human, nematode, bacteria and plant), the utility of the LAF assay in differentiating EVs in pathological settings was assessed, specifically EVs from human cancerous and non‐cancerous natural killer cells. EVs isolated from the NK‐92 cell line (obtained from a patient with aggressive natural killer cell lymphoma) (Klingemann 2023; Gong et al. 1994) and from primary natural killer cells obtained from a healthy donor were characterized and authenticated (Figures 3G–K and S1A). EVs derived from healthy natural killer cells displayed substantially less interactions with VLDL compared to EVs from cancerous natural killer cells (Figure 3L). Additionally, the cancerous natural killer cell EVs also displayed substantially increased binding to VLDL compared to EVs from other non‐cancerous human cells, such as HEK293T embryonic kidney cells and adipose‐derived mesenchymal stromal cells (Figure 3L,F), suggesting that interactions with VLDL may be dependent on disease state. Binding of the cancer cell‐EVs to VLDL also substantially exceeded that of non‐pegylated liposomes (Figures 2G and 3D). Binding was detectable with as few as 5 × 10^6^ EVs and 37 µg/mL of VLDL (protein concentration) from both cancerous and non‐cancerous sources when all other assay parameters remained unchanged, however, the magnitude of the binding signal decreased with lower EV concentrations (data not shown). Taken together, the cancer cell‐derived EVs displayed enhanced binding to VLDL compared to EVs from non‐cancerous cells and synthetic nanoparticles.

### 3.6 LAF Quantifies EV and VLDL Binding in Poorly and Highly Metastatic Contexts

**FIGURE 4:**
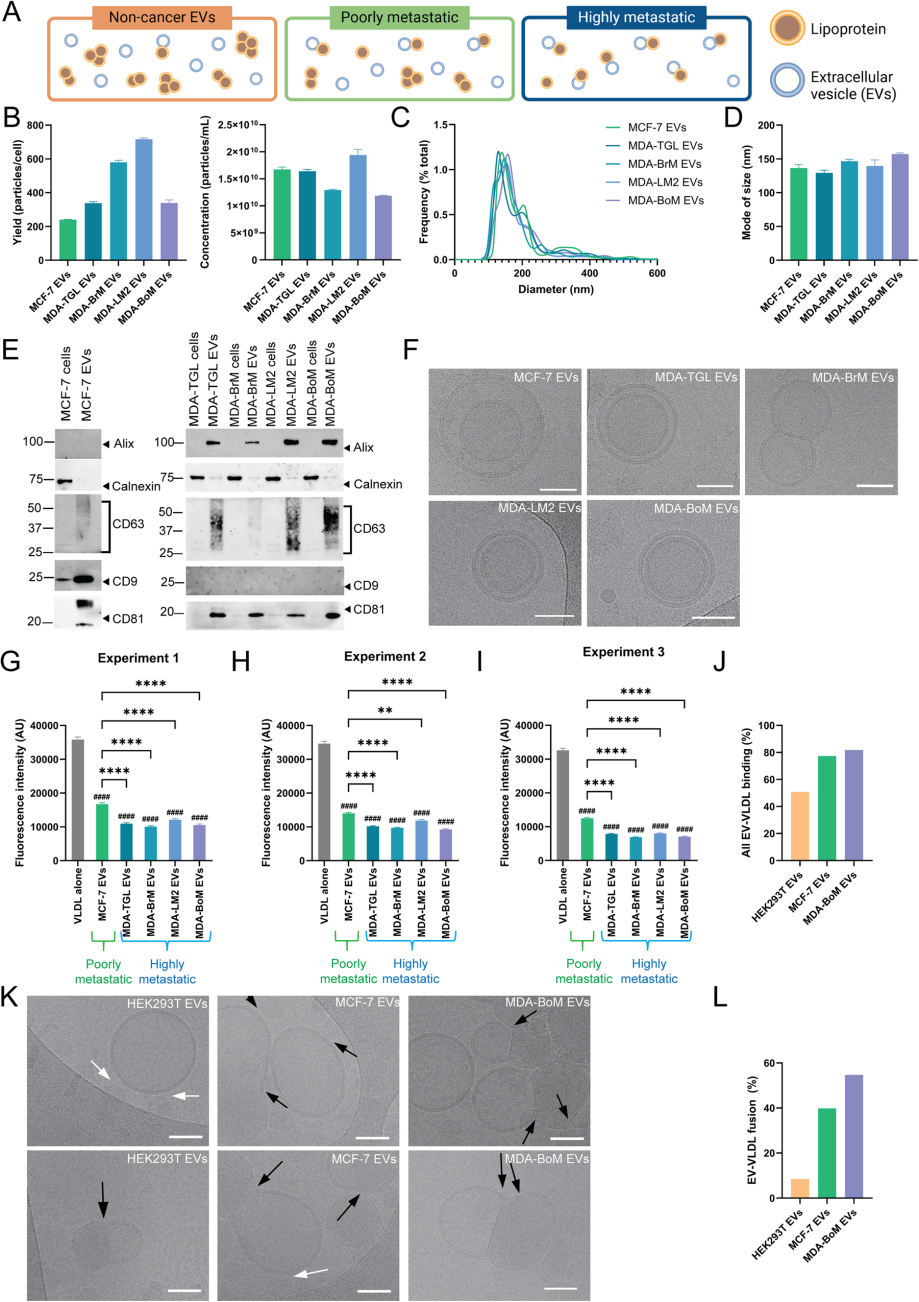
LAF quantifies VLDL binding to EVs from poorly and highly metastatic breast cancer cells. (A) Representative schematic illustration of EV binding to VLDL in non‐cancerous, poorly metastatic and highly metastatic conditions. It should be noted that binding does not always occur on a one‐to‐one basis. Concentration and yield (B), size distribution (C) and mode of size (D) of EVs from MCF‐7 (poorly metastatic breast cancer cell) and highly metastatic breast cancer cells MDA‐MB‐231 TGL (MDA‐TGL), MDA‐MB‐231‐BrM‐831 (MDA‐BrM), MDA‐MB‐231‐BoM‐1833 (MDA‐BoM) and MDA‐MB‐231‐Lm2‐4175 (MDA‐LM2) measured by nanoparticle tracking analysis.Graphs show mean of three representative technical replicates + SEM. Results were validated in at least three separate experiments. (E) Western blot of EV markers (alix, CD63, CD9, CD81) and contaminant marker (calnexin) in EVs and cell homogenates. (F) Cryo‐TEM images of EVs. Scale bar, 50 nm. (G–I) Fluorescence intensity of VLDL/DiI with EVs measured in three separate experiments (reduction indicates binding). (J) Percentage of HEK293T, MCF‐7 and MDA‐MB‐231‐BoM‐1833 EV binding (with and without fusion) with VLDL based on cryo‐TEM images. (K) Representative cryo‐TEM images of binding of EVs to VLDL represented by arrowheads (black: fusion; white: binding without fusion). (L) Percentage of EVs fused with VLDL. The fluorescence intensity of free DiI was subtracted from the data. Bar graphs show the mean of nine technical measurements of one (G) or two (H, I) individual samples + SEM. VLDL alone groups (G–I) display nine technical measurements of one individual sample + SEM. Fluorescent gain settings were maintained across all readings. Statistical analysis performed by ordinary one‐way ANOVA with Tukey's multiple comparison test. ****p < 0.0001; **p < 0.0021 between groups and ^####^ *p* < 0.0001 compared to control.

The finding that EVs from cancerous natural killer cells display increased binding to VLDL compared to EVs from non‐cancerous natural killer cells (Figure 3L), raised the question of whether EV binding to VLDL differs depending on the metastatic potential of the originating cell (Figure 4A). EVs derived from highly metastatic human breast cancer cell lines (MDA‐MB‐231‐TGL, MDA‐MB‐231‐BrM‐831, MDA‐MB‐231‐BoM‐1833 and MDA‐MB‐231‐Lm2‐4175) and a poorly metastatic human breast cancer cell line (MCF‐7) were compared (Figure 4B–F). All breast cancer cell‐derived EVs resulted in a substantial reduction in fluorescence, indicating VLDL binding (Figures 4G–I and S1A). This reduction was more pronounced than any of the EVs from non‐cancerous cells (primary natural killer cells, human embryonic kidney cells and mesenchymal stromal cells) (Figure 3F,L). Notably, the EVs from highly metastatic breast cancer cells displayed a greater reduction in fluorescence intensity compared to EVs from poorly metastatic breast cancer cells (Figure 4G–I). The reproducibility of the LAF assay in identifying differences in EVs from poorly and highly metastatic breast cancer cells is demonstrated in Figure 4G–I, which outlines results obtained on separate occasions. Minimal fluctuations in the absolute fluorescence intensity values across separate experiments indicate the consistency of the LAF assay. Additionally, the reproducibility of the LAF assay was confirmed using multiple VLDL lots supplied by two different commercial vendors (up to three lots from one vendor). Taken together, the LAF assay shows reproducibility in differentiating the binding capacity between VLDL and EVs from highly metastatic versus poorly metastatic breast cancer cells.

Subsequently, cryo‐TEM was used to directly visualize binding between HEK293T EVs, MCF‐7 EVs or MDA‐MB‐231‐BoM‐1833 EVs and VLDL (Figures 4J–L and S1B). Two distinct forms of binding were observed: with fusion (merged phospholipid layers) and without fusion. Among these two forms, fusion is a more reliable indicator of binding, as contact can result from random proximity rather than true binding. Notably, cryo‐TEM images showed that a single EV can associate with multiple VLDL particles, and conversely, a single VLDL particle can bind multiple EVs. The fold difference between the percentage of fusion events was 3.22 (MCF‐7>HEK293T EVs), 4.18 (MDA‐MB‐231‐BoM‐1833>HEK293T EVs) and 1.30 (MDA‐MB‐231‐BoM‐1833>MCF‐7 EVs) (Figure 4L), which correlated to the respective fold differences of 2.96, 3.69 and 0.80, respectively, in the LAF assay (Figures 3F and 4G–I).

**FIGURE 5:**
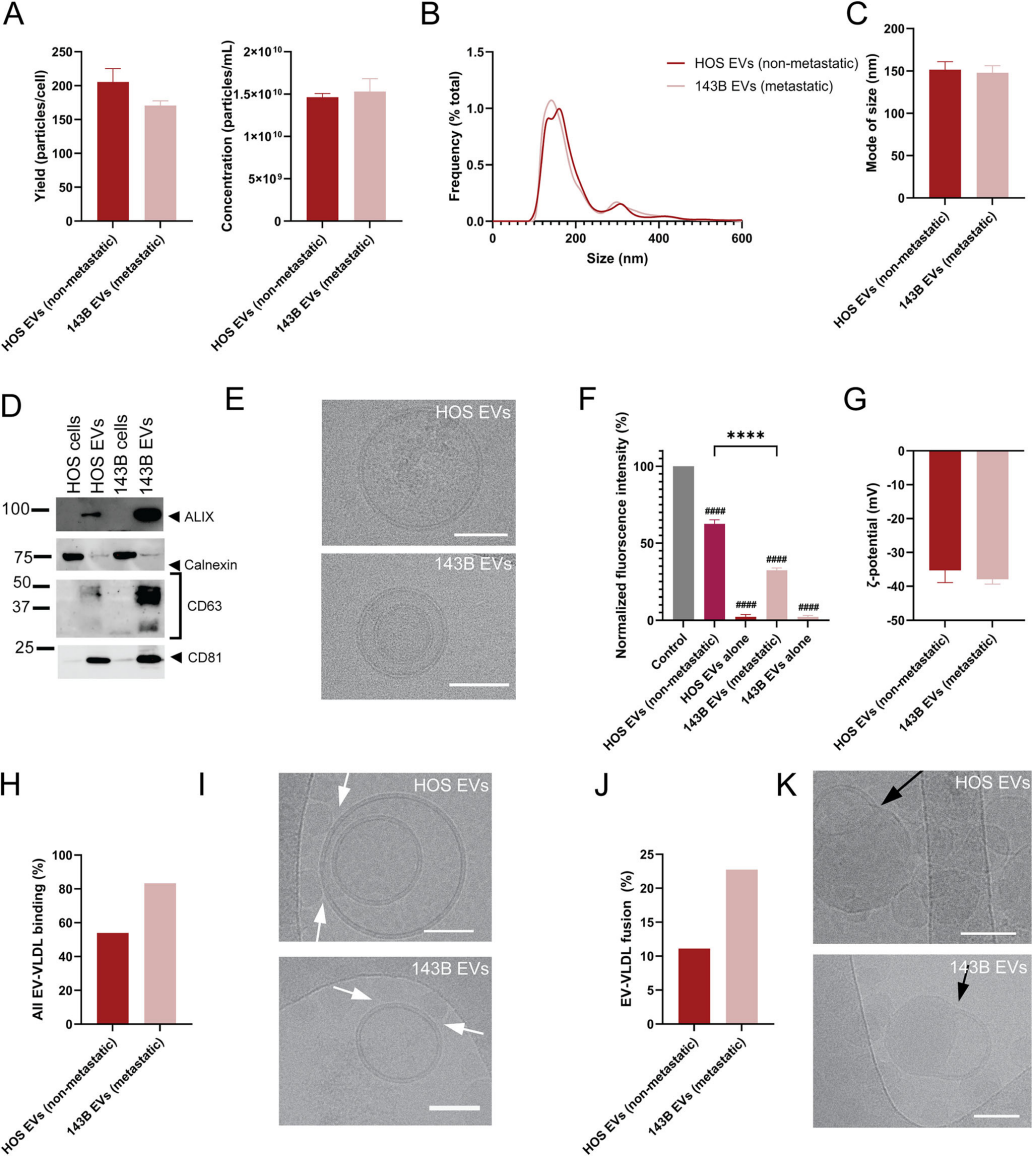
LAF quantifies VLDL binding to EVs from non‐ and highly metastatic patient‐matched osteosarcoma cells. Concentration and yield (A), size distribution (B) and mode of size (C) of EVs derived from non‐metastatic (HOS) and highly metastatic (143B) cells assessed by nanoparticle tracking analysis. (D) Western blot of EV markers (alix, CD63, CD9, CD81) and contaminant marker (calnexin) in EVs and cell homogenate. (E) Cryo‐TEM characterization of EVs. (F) Fluorescence intensity of VLDL/DiI with EVs (reduction indicates binding). (G) Zeta potential of HOS and 143B EVs assessed by laser Doppler microelectrophoresis. (H) Percentage of total EVs binding to VLDL (with and without fusion) based on cryo‐TEM images. (I) Representative cryo‐TEM images of binding of EVs to VLDL (without fusion), as represented by arrowheads. (J) Percentage of EVs fused with VLDL and (K) representative cryo‐TEM images of fusion events. Scale bar, 50 nm. Bar graphs show the mean of three representative measurements of one sample (A‐C, G) or six representative individual samples (F) + SEM. The fluorescence intensity of the free DiI signal was subtracted from the data that was then normalized to the control group (VLDL/DiI alone, 100%). Results were validated in at least three separate experiments. Statistical analysis performed by ordinary one‐way ANOVA with Tukey's multiple comparison test. ****p < 0.0001 between groups and ^####^ *p* < 0.0001 compared to control.

The poorly metastatic breast cancer cell line (MCF‐7) is derived from a different patient and breast cancer subtype than the highly metastatic breast cancer cell line variants (MDA), indicating that the observed differences in EV binding to VLDL may also be due to donor/subtype variability as opposed to metastatic potential of the originating cells. Next, another cancer type (osteosarcoma) was assessed by comparing EVs from non‐metastatic and highly metastatic cancer cells. Notably, in this case, the cells were derived from the same subtype and patient, minimizing genetic variability unrelated to metastatic transformation. Specifically, the non‐metastatic HOS osteosarcoma cell line originates from the same patient as the 143B cell line, which was transformed with an oncogene from Kirsten rat sarcoma virus (v*Kras*), making it metastatic (Luu et al. 2005; Rhim et al. 1975; Rhim et al. 1977; Mcallister et al. 1971). The EVs were authenticated in accordance with the guidelines outlined in the minimal information for studies of EVs (Théry et al. 2018; Welsh et al. 2024) (Figure 5A–E). The patient‐matched non‐metastatic osteosarcoma (HOS) EVs bound substantially less to VLDL than the metastatic osteosarcoma (143B) EVs (Figure 5F), mimicking the pattern seen with VLDL and breast cancer EVs (Figure 4G–I). The difference between VLDL binding to EVs from poorly and highly metastatic cells was more pronounced in the osteosarcoma lines than the breast cancer ones. To confirm that the observed results were not due to the EVs having different interactions with DiI, the fluorescence intensity of the EVs alone was measured and normalized to the VLDL control. Both DiI‐labelled EVs alone had similar negligible fluorescence intensity compared to the samples with VLDL (Figure 5F). Taken together, the findings confirm that the substantial differences in fluorescence intensity observed in the LAF assay were due to varying interactions between EVs and VLDL as opposed to EVs and DiI.

The patient‐matched origin and substantial difference in VLDL binding between HOS and 143B osteosarcoma cells prompted further studies on these EVs. The zeta potentials of the osteosarcoma EVs were compared to assess whether differences in the surface charge of the two EV groups could impact VLDL binding. The results revealed that the zeta potentials of EVs from HOS cells (non‐metastatic) and 143B cells (highly metastatic) were almost identical: −35 mV (HOS) and −38 mV (143B) (Figure 5G), indicating that zeta potential was not a determining factor in VLDL complexation. Cryo‐TEM imaging confirmed that VLDL bound to both HOS and 143B EVs, however, the extent of these associations was greater with the metastatic 143B EVs (Figure 5H,I), mirroring the results of the LAF assay. Several instances of fusion were observed between these EVs and lipoproteins, with a greater number of events present in the 143B EV group (Figures 5J,K and S1B). Fusion with VLDL was 2.05‐fold higher for 143B EVs than for HOS EVs, closely mirroring the 1.81‐fold difference seen in the LAF assay. Taken together, the developed LAF assay revealed that VLDL binds more to EVs from highly metastatic versus poorly metastatic osteosarcoma cells, which was validated with cryo‐TEM.

### 3.7 The LAF Assay Is Broadly Applicable to LDL, HDL and Human Biofluid Samples

**FIGURE 6:**
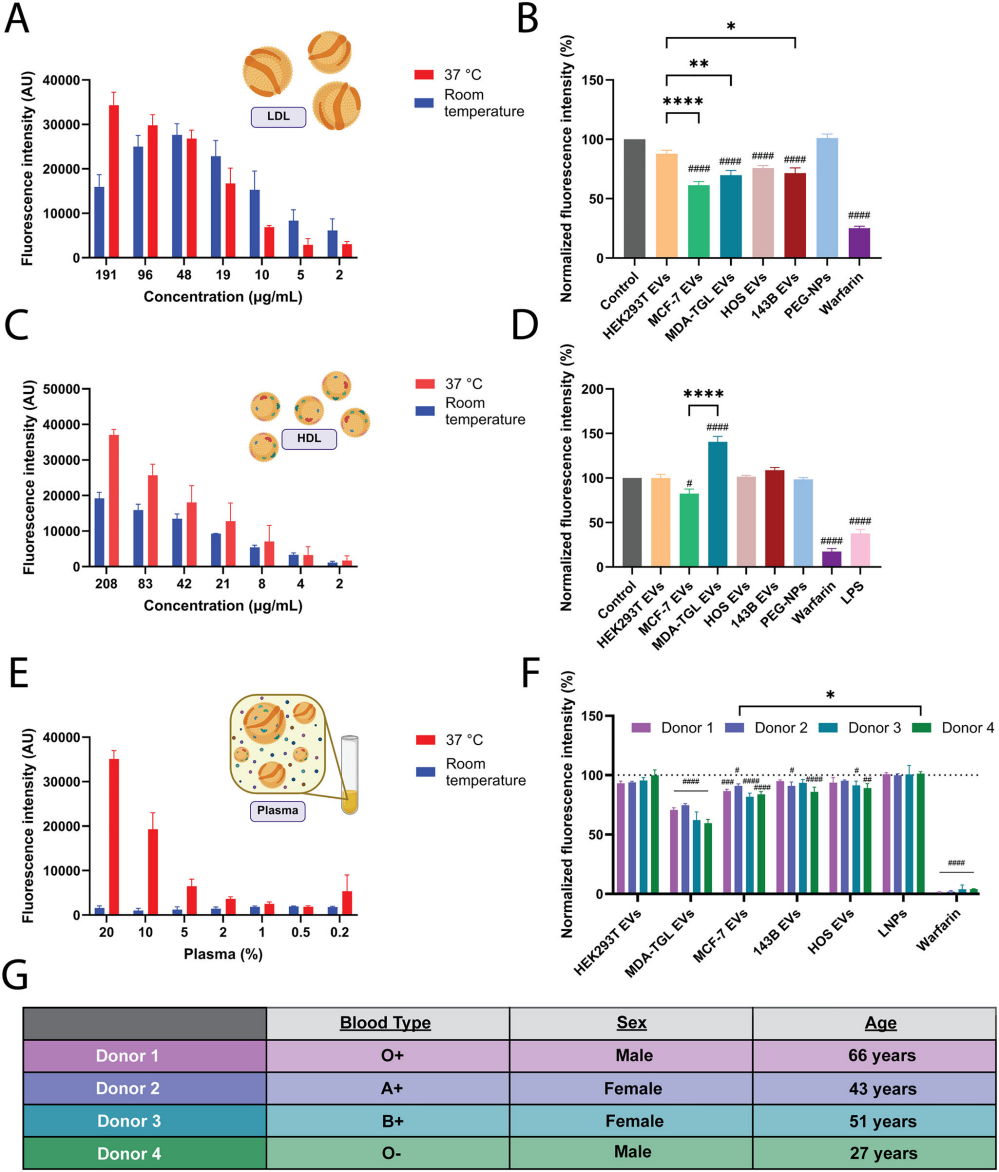
LAF quantifies binding to low‐density lipoprotein (LDL), high‐density lipoprotein (HDL) and lipoproteins in human plasma. (A) Fluorescence intensity of various protein concentrations of DiI‐labelled LDL incubated at 37°C and room temperature for 1 h. (B) Fluorescence intensity of 151 µg/mL LDL (protein concentration) and DiI incubated with warfarin, PEG‐NPs and various EVs (reduction indicates binding). (C) Fluorescence intensity of various protein concentrations of DiI‐labelled HDL incubated at 37°C and room temperature for 1 h. (D) Fluorescence intensity of 37 µg/mL HDL (protein concentration) and DiI incubated with various test agents. (E) Fluorescence intensity of various concentrations of fibrin‐depleted DiI‐labelled plasma incubated at 37°C and room temperature for 1 h. (F) Fluorescence intensity of 20% fibrin‐depleted plasma incubated with test agents. (G) Plasma donor characteristics. Bar graphs show the mean of three representative individual measurements (A, C, E) or six representative individual samples (B, D, F) + SEM. The fluorescence intensity of the free DiI signal was subtracted from the data, which were then normalized to the LDL/DiI, HDL/DiI or plasma/DiI alone control groups (100%). Results were validated in at least three separate experiments. (A, C, E) Fluorescent gain settings were maintained across all readings for non‐normalized data. Statistical analysis performed by ordinary one‐way ANOVA with Tukey's multiple comparison test (B, D) or two‐way ANOVA with Tukey's multiple comparisons test (donors) or Dunnett's multiple comparisons test compared to control (F). ****p < 0.0001; **p < 0.0021; *p < 0.0332 between groups. ^####^ *p* < 0.0001; ^###^ *p* < 0.0002; ^##^ *p* < 0.0021; ^#^ *p* < 0.0332 compared to control. LPS, lipopolysaccharide.

The LAF assay was further expanded to two additional types of lipoproteins: LDL and HDL. Various protein concentrations of LDL were evaluated at 37°C and room temperature (Figure 6A). The fluorescence intensity increased with higher protein concentrations of LDL (Figure 6A). Compared to VLDL (Figure 1B), this increase in fluorescence intensity was less temperature‐dependent (Figure 6A). The LDL protein concentration was optimized to 151 µg/mL at 37°C based on warfarin, which is known to bind to lipoproteins (Rosengren et al. 2012; Yacobi et al. 1976; Tokui et al. 1995). Pegylated nanoparticles and HEK293T EVs showed no significant binding to LDL (Figure 6B). Conversely, non‐metastatic (HOS) and highly metastatic (143B) osteosarcoma EVs, as well as poorly metastatic (MCF‐7) and highly metastatic (MDA‐MB‐231 TGL) breast cancer EVs demonstrated extensive binding to LDL (Figure 6B). Notably, the difference seen in VLDL binding between EVs from poorly and highly metastatic cancer cells (Figures 4G–I and 5F) was not apparent for LDL (Figure 6B).

Similar to LDL, HDL demonstrated a concentration‐dependent increase in fluorescence intensity (Figure 6C) that was less dependent on temperature than VLDL (Figure 1B). A protein concentration of 37 µg/mL and incubation at room temperature was optimized based on known binding to warfarin. LPS, which binds to HDL with high affinity (Tobias et al. 1985; Munford et al. 1981; Levels et al. 2005), was included as an additional control to further validate the LAF assay's ability to detect binding to HDL (Figure 6D). On the contrary to VLDL and LDL, HDL did not display binding to EVs, as the fluorescence intensity did not decrease (Figure 6D). Paradoxically, the highly metastatic breast cancer‐derived EVs (MDA‐MB‐231 TGL) caused the fluorescence intensity to increase beyond temperature/concentration‐dependent effects, suggesting further changes in HDL partitioning triggered by transient binding of MDA‐MB‐231 TGL EVs. In conclusion, we demonstrate that the LAF assay can be expanded to other types of lipoproteins, which show distinct binding patterns (or lack thereof) to EVs.

Next, we sought to determine whether the LAF assay can be used to assess EV binding in complex lipoprotein‐containing biofluids. Human plasma contains various lipoproteins at physiologically relevant concentrations, making it an ideal model to assess the physiological relevance of EV‐lipoprotein binding. Plasma levels above 5% (v/v) displayed markedly higher fluorescence at 37°C than at room temperature (Figure 6E), indicating improved DiI labelling (including likely changes in partitioning) of lipophilic structures. Plasma from four healthy individuals was assessed to account for donor variability. A plasma concentration of 20% at 37°C was optimized based on warfarin binding. Metastatic breast cancer cell‐derived EVs (MDA‐MB‐231 TGL) substantially reduced the fluorescence intensity in all four donors (Figure 6F,G), suggesting binding to lipoproteins. Poorly metastatic breast cancer EVs (MCF‐7) also decreased the fluorescence intensity in all four donors but displayed a less pronounced reduction than MDA‐MB‐231 TGL EVs (Figure 6F). Minimal and donor‐dependent changes were observed when exposing HEK293T EVs, non‐metastatic osteosarcoma (HOS) EVs, highly metastatic osteosarcoma (143B) EVs and pegylated nanoparticles to plasma (Figure 6F). In conclusion, the LAF assay applied to human plasma detected binding and differences in binding of EVs from metastatic and poorly metastatic breast‐cancer‐derived cells (but not from osteosarcoma) across plasma from all four donors, demonstrating donor‐independent affinity despite inherent plasma heterogeneity.

## 4 Discussion

It is important to assess complex formation between EVs and lipoproteins as studies suggest that lipoprotein binding is a critical contributor to the biological identity of EVs, both in terms of cell signalling and transport (Ghebosu et al. 2024). Lipoprotein receptors are abundantly expressed throughout the body in a variety of cell and tissue types (Nguyen et al. 2014; Herz and Hui 2004; Fernández‐Hernando et al. 2009; Kreuter et al. 2002), which may contribute to EV site‐specific targeting and functional effects (Amruta et al. 2023; Iannotta et al. 2024). Interactions between EVs and lipoproteins have been documented in some studies using labour intensive and technically advanced methods (Pham et al. 2023; Busatto et al. 2020; Busatto et al. 2022; Lozano‐Andrés et al. 2023; Sódar et al. 2016). EVs display extensive heterogeneity in terms of surface structure and composition (Rontogianni et al. 2019; Abramowicz et al. 2016; Wiklander et al. 2015; Hoshino et al. 2015), which is likely to affect lipoprotein binding. In previous studies, quantification of EV‐lipoprotein binding and comparisons between various EV types in pathological settings (healthy vs. cancerous; poorly metastatic vs. highly metastatic) have not been assessed due to technical challenges.

Current protocols to assess lipoprotein binding to small molecules consists of methods to separate bound and unbound drug (e.g., equilibrium dialysis, ultrafiltration or ultracentrifugation) followed by a small molecule detection method (e.g., liquid chromatography and mass spectrometry/LC‐MS or high‐performance affinity chromatography/HPAC) (Seyfinejad et al. 2021; Ryu et al. 2019; Eriksson et al. 2005; Bohnert and Gan 2013; Toma et al. 2021). These techniques are time‐consuming and require specialized equipment. Additionally, conventional protocols applied to small molecules are not compatible with synthetic nanoparticles, large biologics and EVs that overlap in size with lipoproteins. Among lipoproteins, VLDL‐like particles bind prominently to EVs (Busatto et al. 2022), which is particularly challenging to assess due to an inability to perform size‐based separation. Therefore, there is a pressing need to develop a broadly applicable, simple, rapid and accessible assay to measure lipoprotein binding.

Here, we present a first‐of‐its‐kind, simple fluorometry‐based LAF assay to rapidly quantify the extent of binding between test agents and fluorescently labelled lipoproteins. Notably, the LAF assay does not rely on size‐based separation and detection of EVs, but rather measures binding‐induced changes in DiI labelling (binding and partitioning) of lipoproteins. EVs from various origins, including human, plant, nematode and bacteria, were assessed in the LAF assay, which was validated with small molecules, proteins, polymers and synthetic nanoparticles that have known binding interactions with lipoproteins. Even in the case of small molecules, the assay outperforms conventional techniques in terms of simplicity and speed. The results demonstrate that the LAF assay is capable of measuring EV interactions with VLDL, LDL and HDL, each of which display distinct EV binding patterns (or lack thereof). Additional validation was performed with cryo‐TEM, which enables visualization and quantification of binding between lipoproteins (lipid monolayer) and EVs (lipid bilayer).

Lipoproteins are also implicated in various (patho)physiological conditions and are capable of aggregating in vitro and in vivo, for example, in atherosclerosis (Guha and Gursky 2011; Guha et al. 2007; Chen et al. 2024; Bhargava et al. 2022; Heffron et al. 2021; Lahelma et al. 2022; La Chica Lhoëst et al. 2025; Öörni and Kovanen 2021). The circulating levels, composition and lipid to protein ratio of lipoproteins vary greatly between individuals and are highly context‐dependent (Lahelma et al. 2022; Kuchinskiene and Carlson 1982; Mittendorfer et al. 2016; Wahl et al. 1981). However, approximations can be made comparing (patho)physiological levels of lipoproteins to those used in the LAF assay (Table 1). In all cases, the concentrations of lipoproteins used in the LAF assay were substantially lower than those in the body. To assess the in vivo relevance of the observed EV‐lipoprotein binding, the LAF assay was also applied to human plasma, which provides a physiologically relevant mixture of lipoproteins.

**TABLE 1:** Comparison of approximate apolipoprotein concentrations used in the LAF assay and (patho)physiological levels.

Lipoprotein and apolipoprotein type	Pathological concentrations	Physiological concentrations	LAF assay concentrations
HDL, apoA‐I	1210 µg/mL (Cole et al. 2025; Holme et al. 2008)	1560 µg/mL Total apoA‐I (Masuda et al. 2023)	24 µg/mL ApoA‐I constitutes approximately 65% of the total protein content in HDL (Bhale and Venkataraman 2022; von Zychlinski et al. 2014)
LDL, apoB‐100	1100 µg/mL (Cole et al. 2025; Holme et al. 2008)	830 µg/mL Total apoB‐100 (in LDL and VLDL) (Masuda et al. 2023)	143.5 µg/mL ApoB‐100 constitutes approximately 95% of the total protein content in LDL (von Zychlinski et al. 2014)
VLDL, apoB‐100	170 µg/mL (Cole et al. 2025)	See above	11.1 µg/mL ApoB‐100 constitutes approximately 30% of the total protein content in VLDL (von Zychlinski et al. 2014)

The findings revealed that HDL displayed minimal binding to EVs, while cancer EVs bound more to LDL than non‐cancerous ones, although differences were not apparent based on the metastatic potential of the originating cells. Additionally, EVs from highly metastatic cancer cell lines displayed increased binding to VLDL/plasma lipoproteins than EVs from non/poorly metastatic ones, suggesting a correlative or causative role of EV/lipoprotein complexes in metastasis. However, in plasma these differences were only observed for breast cancer EVs, while VLDL binding to EVs was distinct based on the metastatic potential of breast cancer and osteosarcoma cells. EV/lipoprotein complexes may have diagnostic potential in predicting the stage of cancer and differentiating cancerous states from healthy ones. The LAF assay may also prove valuable for other disease states in which EVs and lipoproteins are implicated, such as atherosclerosis (Chen et al. 2024; Bhargava et al. 2022; Heffron et al. 2021; Lahelma et al. 2022; Öörni and Kovanen 2021). The presented findings have implications beyond understanding endogenous EVs and developing potential diagnostics. Lipoprotein binding should also be considered for the development of EV therapeutics. Plasma protein binding is used to predict bioavailability, dosing, toxicity and biodistribution of small molecule therapeutics (Seyfinejad et al. 2021; Bohnert and Gan 2013; Di 2021; Porter and Charman 2001). Major drug‐binding components of plasma include lipoproteins (Eriksson et al. 2005; Bohnert and Gan 2013; Di 2021). Lipoproteins have previously been used as drug delivery systems (Busatto et al. 2020; Huang et al. 2015) and identified as targets to enhance the permeability and delivery of nanomedicines (Jiang et al. 2022). Studies have demonstrated that binding and functionalization of synthetic nanoparticles with the protein components of lipoproteins (apolipoproteins) can promote blood‐brain barrier crossing (Prawatborisut et al. 2022; Kreuter et al. 2002; Neves et al. 2017).

In the future, it will be important to identify biomolecular surface compositions and physical membrane characteristics of EVs that promote complex formation with lipoproteins and their relation to homeostasis and disease pathology. It is important to note that while the LAF assay delivers robust signals, it infers lipoprotein‐EV binding indirectly via DiI, and therefore, remains semi‐quantitative, without yielding absolute binding values. Additionally, the assay is affected by the source of lipoprotein/plasma, which likely differs based on the donor, supplier and batches. Therefore, optimal assay conditions may need to be assessed for each batch using a positive control of binding (warfarin).

## Author Contributions

**Raluca Ghebosu**: conceptualization, (lead), formal analysis, (lead), funding acquisition, (supporting), investigation, (lead), methodology, (lead), visualization, (lead), writing ‐ review & editing, (lead). **Jenifer Pendiuk Goncalves**: methodology, (supporting), resources, (supporting), supervision, (supporting), writing ‐ review & editing, (supporting). **Nur Indah Fitri**: investigation, (supporting), validation, (supporting), writing ‐ review & editing, (supporting). **Dalila Iannotta**: investigation, (supporting), resources, (supporting), writing ‐ review & editing, (supporting). **Mohammad Farouq Sharifpour**: methodology, (supporting), resources, (supporting), Writing ‐ review & editing, (supporting). **Elaina Coleborn**: funding acquisition, (supporting), resources, (supporting), writing ‐ review & editing, (supporting). **Alex Loukas**: funding acquisition, (supporting), supervision, (supporting), writing ‐ review & editing, (supporting). **Fernando Souza‐Fonseca‐Guimaraes**: funding acquisition, (supporting), resources, (supporting), supervision, (supporting), writing ‐ review & editing, (supporting). **Joy Wolfram**: conceptualization, equal, funding acquisition, (lead), project administration, (lead), supervision, (lead), writing ‐ original draft, (equal), writing ‐ review & editing, (supporting)

## Conflicts of Interest

J.W. is or has been a board member or scientific advisor of biomedical companies: Omnidermal, Genomill and Pharmatest Services. Ionis Pharmaceuticals and Sartorius have sponsored or are sponsoring research in J.W.’s laboratory. J.W. and R.G. have submitted a provisional patent (‘Plasma protein binding and uses thereof,’ reference number: 2025901353) on this technology.

## Supporting information



Supplementary Figure 1. Low magnification cryogenic transmission electron microscopy (cryo‐TEM) images of extracellular vesicles (EVs).

## Data Availability

The raw data that support the findings will be available upon reasonable request by contacting the corresponding author.
