# Supplementary material for: Lipoprotein Association Fluorometry (LAF) as a Semi‐Quantitative Characterization Tool to Assess Extracellular Vesicle‐Lipoprotein Binding
Source: J Extracell Vesicles. 2025 Sep 30;14(10):e70172. doi: 10.1002/jev2.70172 (PMC12481431; doi:10.1002/jev2.70172)
Supplement: Supplementary file 1 — Supplementary Figure 1. Low magnification cryogenic transmission electron microscopy (cryo‐TEM) images of extracellular vesicles (EVs). [file JEV2-14-e70172-s001.docx]

Supplementary Materials for

**Lipoprotein association fluorometry (LAF) as a semi-quantitative characterization tool to assess extracellular vesicle-lipoprotein binding**

Raluca Ghebosu, Jenifer Pendiuk Goncalves, Nur Indah Fitri, Dalila Iannotta, Mohammad Farouq Sharifpour, Elaina Coleborn, Alex Loukas, Fernando Souza-Fonseca-Guimaraes, Joy Wolfram^*^

*Corresponding author: j.wolfram@uq.edu.au (JW)

**This file includes:**

Figure S1


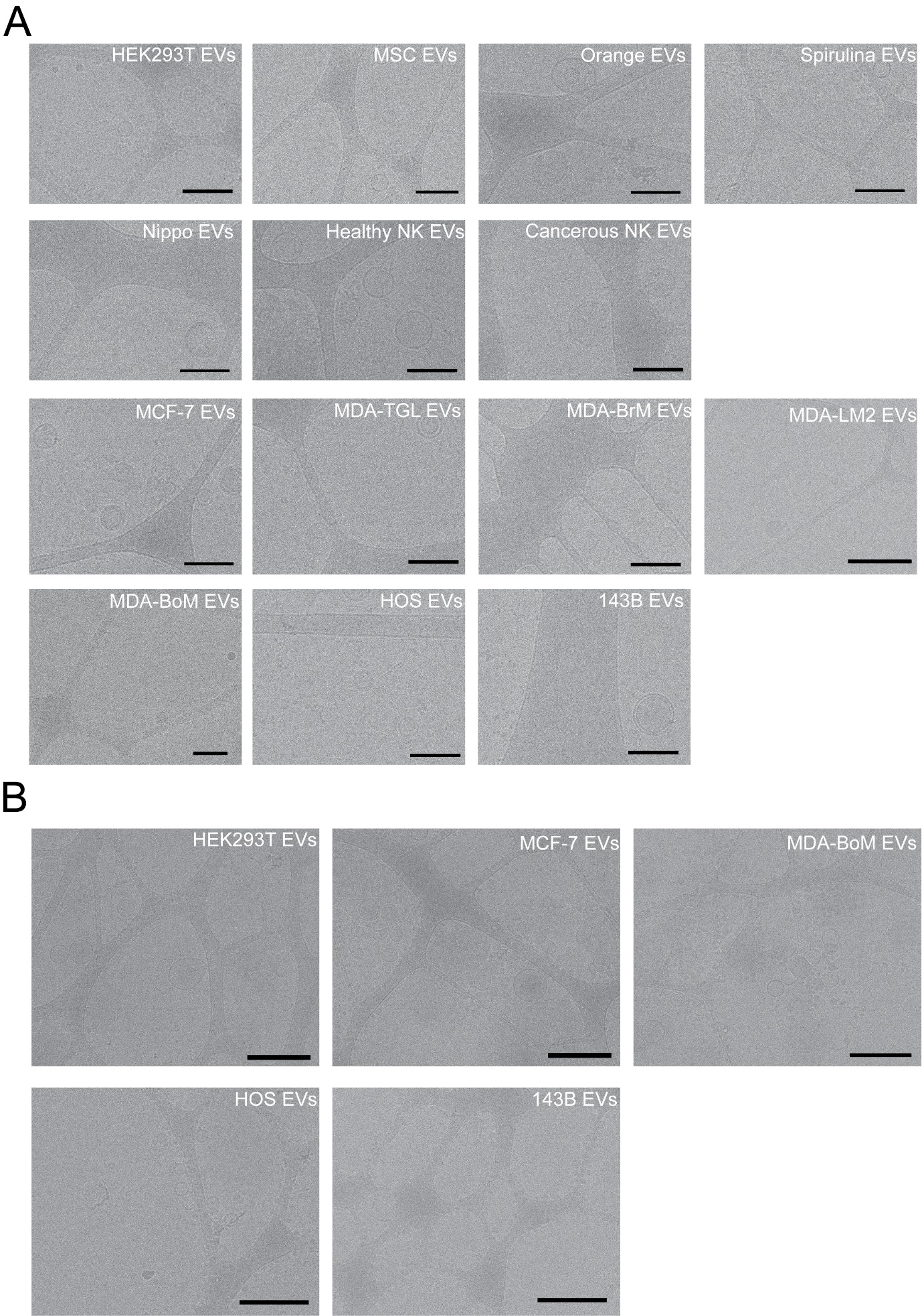


**Supplementary Figure 1. Low magnification cryogenic transmission electron microscopy (cryo-TEM) images of extracellular vesicles (EVs).** (A) Low magnification cryo-TEM of various EVs utilized in the lipoprotein association fluorometry (LAF) assay. Scale bar, 250 µm. (B) Low magnification images of very-low-density lipoprotein (VLDL) binding to various EVs. Scale bar, 500 µm.
